# Comprehensive Evaluation of the Association of *APOE* Genetic Variation with Plasma Lipoprotein Traits in U.S. Whites and African Blacks

**DOI:** 10.1371/journal.pone.0114618

**Published:** 2014-12-12

**Authors:** Zaheda H. Radwan, Xingbin Wang, Fahad Waqar, Dilek Pirim, Vipavee Niemsiri, John E. Hokanson, Richard F. Hamman, Clareann H. Bunker, M. Michael Barmada, F. Yesim Demirci, M. Ilyas Kamboh

**Affiliations:** 1 Department of Human Genetics, Graduate School of Public Health, University of Pittsburgh, Pittsburgh, Pennsylvania, United States of America; 2 Department of Epidemiology, Colorado School of Public Health, University of Colorado Denver, Aurora, Colorado, United States of America; 3 Department of Epidemiology, Graduate School of Public Health, University Pittsburgh, Pittsburgh, Pennsylvania, United States of America; McMaster University, Canada

## Abstract

Although common *APOE* genetic variation has a major influence on plasma LDL-cholesterol, its role in affecting HDL-cholesterol and triglycerides is not well established. Recent genome-wide association studies suggest that *APOE* also affects plasma variation in HDL-cholesterol and triglycerides. It is thus important to resequence the *APOE* gene to identify both common and uncommon variants that affect plasma lipid profile. Here, we have sequenced the *APOE* gene in 190 subjects with extreme HDL-cholesterol levels selected from two well-defined epidemiological samples of U.S. non-Hispanic Whites (NHWs) and African Blacks followed by genotyping of identified variants in the entire datasets (623 NHWs, 788 African Blacks) and association analyses with major lipid traits. We identified a total of 40 sequence variants, of which 10 are novel. A total of 32 variants, including common tagSNPs (≥5% frequency) and all uncommon variants (<5% frequency) were successfully genotyped and considered for genotype-phenotype associations. Other than the established associations of APOE*2 and APOE*4 with LDL-cholesterol, we have identified additional independent associations with LDL-cholesterol. We have also identified multiple associations of uncommon and common *APOE* variants with HDL-cholesterol and triglycerides. Our comprehensive sequencing and genotype-phenotype analyses indicate that *APOE* genetic variation impacts HDL-cholesterol and triglycerides in addition to affecting LDL-cholesterol.

## Introduction

Coronary heart disease (CHD), a multifactorial disease modulated by multiple genetic and environmental factors, continues to be a leading cause of morbidity and mortality worldwide [1]. Dyslipidemia with high low-density lipoprotein cholesterol (LDL-C) and low high-density lipoprotein cholesterol (HDL-C) is associated with high risk of CHD [1]. Genes involved in lipid metabolism are considered to be candidate genes for CHD risk, and their genetic variation could contribute, in part, to the inter-individual variation in plasma lipoprotein-lipid levels.

Apolipoprotein E (ApoE, protein; *APOE*, gene) is a major constituent of very low-density lipoproteins (VLDL) and high-density lipoproteins (HDL) [2], [3] and plays a crucial role in lipid metabolism through enhancing hepatic uptake of triglyceride-rich lipoproteins (TGRL) and participating in reverse cholesterol transport mechanism (RCT) [4]. Besides its significant contribution in lipid metabolism, ApoE is involved in multiple functions in the human body, including nerve growth and regeneration [5]–[8], cognitive function [9], [10], immunoregulation and influencing susceptibility to infectious diseases [11]–[13]. The *APOE* gene is located on chromosome 19q13.32 as part of the *APOE-C1-C4-C2* gene cluster, and is composed of 4 exons and 3 introns that span 3.6 kb [14] and encodes for 299 amino acids [3].

*APOE* is one of the most extensively studied candidate genes and the influence of its genetic variation on plasma lipid levels and CHD risk has been well investigated [15]–[16]. The epsilon polymorphism of *APOE* is defined by the rs7412 and rs429358 SNPs which leads to the generation of ApoE2, ApoE3 and Apo E4 isoforms and are coded by three codominant alleles (designated as E*2 E*3 and E*4). The three isoforms differ by an amino acid substitution at position 112 or position 158 in the 299-amino-acid peptide chain. Although the major effect of *APOE* genetic variation has been reported to be on LDL-C levels, recent genome-wide association studies (GWAS) on lipid traits also identified statistically significant associations of *APOE* common variants with HDL-C and triglyceride (TG) levels [17]–[18]. Thus, deep resequencing of the *APOE* gene in selected individuals with high/low lipid levels is warranted in order to characterize both rare and common variants that might affect plasma lipid profile.

In this study, we resequenced the entire *APOE* gene region (total 5.5 kb), including all four exons (1,180 bp), three introns (2,432 bp), and ∼1 kb of each of the flanking regions in selected individuals with extreme HDL-C levels (falling within the upper and lower 10^th^ percentiles) from two ethnically-distinct populations (95 US non-Hispanic Whites (NHWs) and 95 African Blacks). Following the sequencing-based discovery step, we genotyped all identified common tagSNPs (*r^2^*≥0.9) with minor allele frequency (MAF) ≥5%, and relevant uncommon and rare variants with MAF<5% in the entire sample sets (623 NHWs and 788 African Blacks) to evaluate their associations with lipid traits. The association of *APOE* genetic variation was examined with three lipid traits (LDL-C, HDL-C and TG) and apolipoprotein B (ApoB) using single-site association analysis for variants with MAF≥1%, gene–based and haplotype-based association analyses for all variants, and SKAT-O (sequencing Kernel association optimal test) for uncommon and rare variants (MAF<5%).

## Materials and Methods

### Study Samples

The study was conducted on two epidemiologically well-characterized population samples comprising 623 US non-Hispanic Whites (NHWs) and 788 African Blacks. NHW samples were collected as part of the San Luis valley Diabetes Study that was designed as geographical case-control study of non-insulin dependent diabetes mellitus and cardiovascular disease in Alamosa and Conejos counties of South Colorado [19). All NHWs used in this study were non-diabetic controls and the basic characteristics of this study are described elsewhere [19]–[20]. African Blacks were recruited from Benin City, Nigeria as part of a study on CHD risk factors in Blacks and the study details have been described in Bunker et al. [21]–[22]. While LDL-C, HDL-C and TG were measured in all subjects, ApoB was measured only in a subset of NHW individuals [23]–[24]. The demographic and lipid characteristics of these study samples can be found in our previous publications [24]–[26]. The study was approved by the University of Pittsburgh and University of Colorado Denver Institutional Review Boards and all study participants provided written informed consent.

### DNA Extraction

The genomic DNA used for sequencing and genotyping was extracted from blood clots in Blacks and from buffy coats in NHWs using standard procedures.

### DNA Sequencing

Ninety-five individuals with high HDL-C levels falling within the upper 10^th^ percentile (47 NHWs, and 48 African Blacks) and 95 individuals with low HDL-C levels falling in the lower 10^th^ percentile (48 NHWs, and 47 African Blacks) were selected for Sanger sequencing. The characteristics of the selected samples in both ethnic groups are summarized in **Table S1 in S1 File**.

A total of ∼5.5 kb of the *APOE* gene region, including all 4 exons and 3 introns, 1,034 bp in 5′ flanking region, and 845 bp in 3′ flanking region were PCR-amplified using M13 tagged forward and reverse primers. Publicly available information at SeattleSNPs database (http://pga.mbt.washington.edu/) was used to order M13 tagged primers, which generated nine overlapping PCR amplicons. PCR reaction and cycling conditions are available upon request. The PCR-amplified samples were sent to a commercial lab (Beckman Coulter Genomics, Danvers, MA) for automated fluorescence-based cycle sequencing and capillary electrophoresis on ABI 3730x1DNA Analyzers. Variant Reporter version 1.0 (Applied Biosystems, Foster City, CA) and Sequencher version 4.8 (Gene Codes Corporation, Ann Arbor, MI) were used for sequencing analysis and variant detection.

### DNA Genotyping

Common tagSNPs (MAF≥5%) were determined by Tagger analysis of the sequencing data in each ethnic group using Haploview software and an *r^2^* cut-off of 0.9. All common tagSNPs and uncommon/rare variants (MAF<5%) identified in each ethnic group by our sequencing, as well as the suspicious variants with low sequencing quality and/or low coverage that warrant validation and the previously reported common variants not detected in our sequencing, were selected for follow-up genotyping.

TaqMan (Applied Biosystems) or iPLEX Gold (Sequenom, San Diego, CA) genotyping methods were used for genotyping following manufacturer's protocols and recommendations. Whole genome amplified DNAs dried in 384-well plates were used for genotyping. Endpoint fluorescence reading of custom or pre-made TaqMan assays was done using the ABI Prism 7900HT Sequence Detection System. The iPLEX Gold genotyping was performed in the Core laboratories of the University of Pittsburgh. Sequences of primers and probes used for genotyping are available upon request. All the samples used in sequencing were also included in genotyping as a quality control measure. The comparison of sequencing and genotyping calls was conducted to check the concordance as well as to increase the call rate in both sequencing and genotyping sets.

### Statistical Analysis

Analyses for NHWs and African Blacks were performed separately. For sequencing subsets, the Haploview software (www.broadinstitute.org/haploview) was used to analyze allele frequencies, their distributions in the two extreme HDL-C groups, their concordance with Hardy-Weinberg Equilibrium (HWE), and their linkage disequilibrium (LD) patterns.

SNPs with extensive missing data (>20%) and/or deviating highly from HWE (P<0.01) were excluded from association analyses. A total of 15 variants in NHWs and 23 variants in Blacks remained for downstream analysis. The associations between SNPs and lipid traits were analyzed using additive linear regression model. We took the best power Box-Cox transformation such that the transformed lipid traits achieved normality. Stepwise regression in both directions was performed to identify significant covariates for each lipid trait. The covariates included were gender, age, BMI and smoking in NHWs and gender, age, BMI, waist measurement, smoking, exercise (minutes walking or bicycling to work each day), and staff level (junior or senior) in Blacks. Detailed information on those covariates and their effects can be found elsewhere [24]. Since the epsilon *APOE E2/E3/E4* polymorphism has an established effect on cholesterol levels, we also adjusted the effects of novel associations for the epsilon *APOE* polymorphism. Single-site, haplotype-based and rare variants analyses were implemented in R and the versatile gene-based associations (VEGAS) [27] were also performed. For single-site analysis, we applied Benjamini-Hochberg procedure [28] to control for false discovery rate (FDR) and considered an FDR (q-value) of <0.20 as statistically significant.

For haplotype association analysis, the generalized linear model (GLM) was used [29]. Including too many haplotypes can make above model inefficient and impractical. To reduce the number of haplotypes considered in association analysis, we used the sliding window, 4 SNPs per window, and assessed evidence for association within each window. Specifically, a global p-value for testing overall effects of the haplotypes with frequency greater than 0.01 was used to assess the associations between the traits and haplotypes in each window. Sliding-window haplotype analysis was performed using the haplo.glm function in the Haplo.Stats R package (version 1.5.0).

We analyzed the cumulative effects of uncommon/rare variants by using the SKAT-O method [30], which has been proposed to be the optimal test for rare variant analysis and outperformed the SKAT and burden tests in several ways. The analysis was performed by using three different minor allele frequency bin thresholds (≤1%, ≤2% and <5%). The SKAT method was implemented using the “SKAT” R package.

## Results

### *APOE* Sequencing Results

Sequencing of ∼5.5 kb genomic region of *APOE* (including all 4 exons, 3 introns, 1,034 bp in the 5′ and 845 bp in the 3′ flanking regions), in 190 selected individuals (95 NHWs and 95 African Blacks) with extreme HDL-C levels revealed a total of 40 variants in both population groups, including 30 known and 10 novel variants (as compared to NCBI dbSNP human Build 141) (**Table S2 in S1 File**). All novel variants identified in this study have been submitted to dbSNP database: (http://www.ncbi.nlm.nih.gov/SNP/snp_viewTable.cgi?handle=KAMBOH).

The codon position used for specifying the coding variants corresponds to the premature protein that also includes the first 18 amino acids of signal peptide. The distribution of the 40 variants is as follows: 10 in 5′ flanking region, 7 in exons (including 2 in 3′ UTR), 16 in introns (including 1 in splice site), and 7 in the 3′ flanking region. Four of the 5 coding variants (80%) were non-synonymous. Ten of the 40 variants were present in both groups, while 9 variants were unique to NHWs and 21 variants were specific to African Blacks. Four of the ten shared-variants showed statistically significant allele frequency differences between the two ethnic groups (see **Table S2 in S1 File** for variants at positions 560, 624, 832, and 1163).

### Distribution of *APOE* sequence variants in two extreme HDL-C groups

Comparison of sequencing variants distribution between the two extreme HDL-C groups in NHWs and African Blacks is presented in **Table S3 in S1 File** and **Table S4 in S1 File**, respectively.

Among the 8 rare/uncommon variants (overall MAF<5%) in NHWs, 6 were unique to the high HDL-C group, 1 was unique to the low HDL-C group, and 1 was present in both lipid groups. In parallel with observing more unique rare variants in the high HDL-C group, 21% (10/47 subjects) of this group had at least one unique rare variant as compared to 2% (1/48 subjects) of the other lipid group (Fisher exact test p-value = 0.0037). Furthermore, the two rare coding variants observed in this study (Ala23Ala; Val254Glu) were present only in the high HDL-C group.

Among the 21 rare/uncommon variants (overall MAF<5%) observed in African Blacks, 6 were unique to the high HDL-C group, 5 were unique to the low HDL-C group, and the remaining 10 were equally distributed among the two extreme HDL-C groups. Unlike NHWs, the distribution of the unique rare variants was similar in the two extreme lipid groups among African Blacks. Fifteen percent (7/48 subjects) of the high HDL-C group had at least one unique rare variant as compared to 6% (3/47 subjects) of the low HDL-C group (Fisher exact test p-value = 0.316).

### Single-site association analysis of the SNPs in the entire NHW and Black samples

Following the identification of genetic variation in the sequencing step, common tagSNPs covering the entire *APOE* gene and rare variants were genotyped in the total sample of NHWs (n = 623) and African Blacks (n = 788) for genotype-phenotype association analyses. Initially, 20 variants in NHWs (9 tagSNPs, 8 rare variants, 2 suspicious SNPs, and 1 database SNP) and 32 variants in African Blacks (9 tagSNPs, 21 rare variants, 1 suspicious SNP, and 1 database SNP) were selected for genotyping. In NHWs, 2 of the 20 variants (*APOE*2294; MAF = 0.005, and *APOE*4951/rs1081105;MAF = 0.042) failed in both Sequenom and TaqMan designs or runs, 2 suspicious variants (*APOE*4489, and *APOE*4490) were confirmed as not being genuine and one variant (*APOE*624/rs769446) with low call rate was excluded from the association analyses. So, a total of 15 variants (14 sequencing variants and 1 database SNP *APOE*3106/rs769452) were successfully genotyped in the entire NHW sample. In African Blacks, 6 of 32 variants (*APOE*471/rs439382;MAF = 0.132, *APOE*494;MAF = 0.005, *APO*E526;MAF = 0.005, *APOE*2576;MAF = 0.005, *APOE*4951/rs1081105; MAF = 0.042, and *APO*E5229/rs80125357; MAF = 0.059) failed in both Sequenom and TaqMan designs or runs, the database SNP (*APOE*1586/rs74625294) and the suspicious variant (*APOE*91) were excluded because they turned out to be non-polymorphic in our population and an additional variant (*APOE*1591/rs147236548) was excluded from the statistical analyses because it was out of HWE. Thus, a total of 23 variants were successfully genotyped in the entire African Black sample.

**Figure 1:**
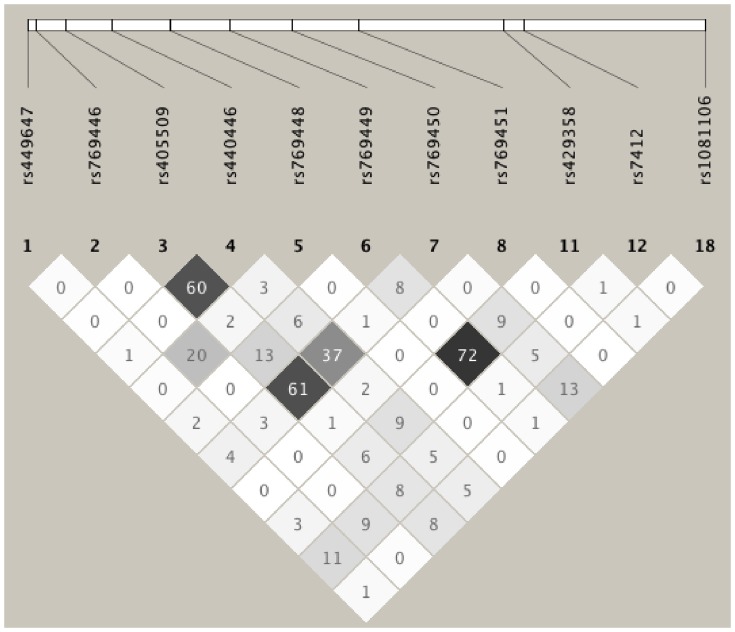
LD Plot of the genotyped variants with MAF>1% in NHWs. The values in the cells are the pairwise degree of LD indicated by *r^2^*×100. *r* ^2^ = 0 is shown as white, 0<*r* ^2^<1 is shown in gray and *r* ^2^ = 1 is shown in black.

The LD plot of the genotyped variants with MAF>1% in NHWs is shown in **Fig. 1**, the association results for all genotyped variants with the three lipid traits (LDL-C, TG, and HDL-C) and ApoB are presented in **Table 1** and the adjusted mean distributions of all the evaluated lipid traits among the genotype groups are given in **Table S5 in S1 File**. As expected, the two known and well-established SNPs as part of the *APOE* epsilon polymorphism, E*4 (rs429358) and E*2 (rs7412) were significantly associated with plasma levels of LDL-C (β = 8.10; p = 0.0103, and β = −21.84; p = 1.84E-07, respectively) and ApoB (β = 2.14; p = 0.0005, and β = −5.60; p = 9.65E-13, respectively). Four additional LDL-C associations were observed independent of E*2/E*4: *APOE*832/rs405509 in 5′ flanking (β = −5.17; p = 0.0345; FDR = 0.139), *APOE*1163/rs440446 in intron 1 (β = 6.11; p = 0.018; FDR = 0.139), *APOE2*440/rs769450 in intron2 (β = 5.52; p = 0.0275; FDR = 0.139), and *APO*E4310/rs199768005 (Val254Glu) in exon4 (β = −35.36; p = 0.043; FDR = 0.139). These same four SNPs were also associated with TG (p = 0.0019 and FDR = 0.01, p = 0.0012 and FDR = 0.01, p = 0.002 and FDR = 0.01, and p = 0.028 and FDR = 0.074, respectively). An additional SNP, *APO*E4528/rs374329439 in 3′UTR, was also associated with TG (p = 0.022; FDR = 0.071).

**Table 1:** Single-site association analysis with lipid traits in NHWs.

	Variant					LDL-C					ApoB^a^					TG^a^					HDL-C^a^				
								*E* * *2*/*E* * *4* Adjusted Result					*E* * *2*/*E* * *4* Adjusted Result					*E* * *2*/*E* * *4* Adjusted Result					E2/E4 Adjusted Result		
Nucleotide position	Chr. Position	MAF	HWE-P	Location	RegulomeDB score	Beta	P	Adj. Beta	Adj. P	FDR	Beta	P	Adj. Beta	Adj. P	FDR	Beta	P	Adj. Beta	Adj. P	FDR	Beta	P	Adj. Beta	Adj, P	FDR
*APOE*560/rs449647	44905307	0.161	0.297	5′ flanking	5	−7.12	**0.0247**	−0.89	0.7922	0.887	−0.95	0.1290	0.40	0.5347	0.926	−0.020	0.5229	−0.022	0.5342	0.630	−0.01	0.3526	−0.010	0.5568	0.724
*APOE*832/rs405509	44905579	0.4775	1	5′ flanking	1f	0.42	0.8542	−5.17	**0.0345**	**0.139**	1.46	**0.0009**	−0.06	0.9052	0.926	−0.070	**0.0033**	−0.079	**0.0019**	**0.010**	0.01	0.4031	0.010	0.3963	0.724
*APOE*1163/rs440446	44905910	0.3604	0.434	Intron 1	4	−2.59	0.2810	−6.11	**0.0180**	**0.139**	0.56	0.2319	−0.17	0.7387	0.926	−0.080	**0.0018**	−0.088	**0.0012**	**0.010**	0.01	0.2865	0.008	0.5280	0.724
*APOE*1575/rs769448	44906322	0.021	0.235	Intron 1	4	0.75	0.9232	−1.07	0.8869	0.887	0.94	0.5204	0.37	0.7901	0.926	−0.090	0.2323	−0.094	0.2333	0.505	0.08	**0.0197**	0.083	**0.0223**	0.291
*APOE*1998/rs769449	44906745	0.1165	0.437	intron 2	4	6.95	0.0551	1.31	0.8438	0.887	2.03	**0.0030**	−0.68	0.5963	0.926	0.020	0.6867	0.042	0.5468	0.630	−0.01	0.5800	0.009	0.7805	0.846
*APOE*2440/rs769450	44907187	0.4015	0.452	intron 2	5	4.89	**0.0378**	5.52	**0.0275**	**0.139**	0.15	0.7422	0.39	0.4242	0.926	0.060	0.0082	0.080	**0.0022**	**0.010**	0.003	0.7725	−0.005	0.6739	0.796
*APOE*2907/rs769451	44907654	0.0112	1	intron 2	5	0.45	0.9668	−5.32	0.6255	0.887	−0.22	0.9196	−0.52	0.8129	0.926	−0.050	0.6157	−0.083	0.4653	0.630	0.05	0.3390	0.054	0.3059	0.663
*APOE*3038/rs111833428	44907785	0.0016	1	exon 3 (Ala 23 Ala)	5	27.34	0.3354	25.51	0.3533	0.657	13.81	**0.0342**	13.27	**0.0312**	0.374	0.160	0.5766	0.166	0.5650	0.630	0.10	0.4366	0.095	0.4759	0.724
*APOE*3106/rs769452	44907853	0.0008	1	exon 3 (Leu46Pro)	5	18.03	0.6522	11.16	0.7739	0.887	9.15	0.1602	7.28	0.2390	0.926	−0.220	0.5865	−0.225	0.5813	0.630	0.20	0.2787	0.214	0.2544	0.661
*APOE*3937/rs429358(*E* * *4*)	44908684	0.1525	1	exon 4 (Cys 130 Arg)	5	8.10	**0.0103**				2.14	**0.0005**				0.010	0.7069				−0.02	0.2233			
*APOE*4075/rs7412(*E* * *2*)	44908822	0.0806	0.788	exon 4 (Arg 176 Cys)	5	−21.84	**1.84E-07**				−5.60	**9.65E-13**				0.010	0.7438				−0.02	0.4524			
*APOE*4310/rs199768005	44909057	0.004	1	exon 4 (Val 254 Glu)	5	−32.56	0.0705	−35.36	**0.0427**	**0.139**	1.05	0.822	−0.41	0.9260	0.926	−0.400	**0.0260**	−0.400	**0.0283**	**0.074**	0.01	0.8927	0.004	0.9619	0.962
*APOE*4528/rs374329439	44909275	0.0008	1	exon 4 (3′ UTR)	5	−12.30	0.7585	−14.32	0.7119	0.887	NA*	NA*	NA*	NA*	NA*	0.920	**0.0232**	0.930	**0.0218**	**0.071**	0.24	0.1926	0.236	0.2085	0.661
*APOE*4737/rs117656888	44909484	0.0081	1	3′ flanking	5	13.58	0.2867	11.91	0.3370	0.657	1.60	0.52	1.23	0.6031	0.926	0.090	0.5084	0.092	0.4795	0.630	0.12	**0.0487**	0.110	0.0676	0.439
*APOE*5361/rs1081106	44910109	0.0852	1	3′ flanking	3a	4.00	0.3272	3.77	0.3537	0.657	0.12	0.8813	0.48	0.5570	0.926	−0.006	0.8928	−0.007	0.8638	0.864	0.03	0.0915	0.028	0.1571	0.661

* (NA) unavailable results because of missing phenotype data for subjects who carry the rare allele, Nucleotide position is according to the reference sequence (Accession # AF261279.1); Chr. Position: chromosomal position is according to NCBI dbSNP human Build 141. ^a^Box-Cox transformed variables. MAF: minor allele frequency. HWE-P: Hardy Weinberg equilibrium p-value.

RegDB scores: RegulomeDB scores. LDL-C: low-density lipoprotein cholesterol; ApoB: Apolipoprotein B; TG: triglyceride; HDL-C: high-density lipoprotein cholesterol. **Bold** values represent significant p-values

**Figure 2:**
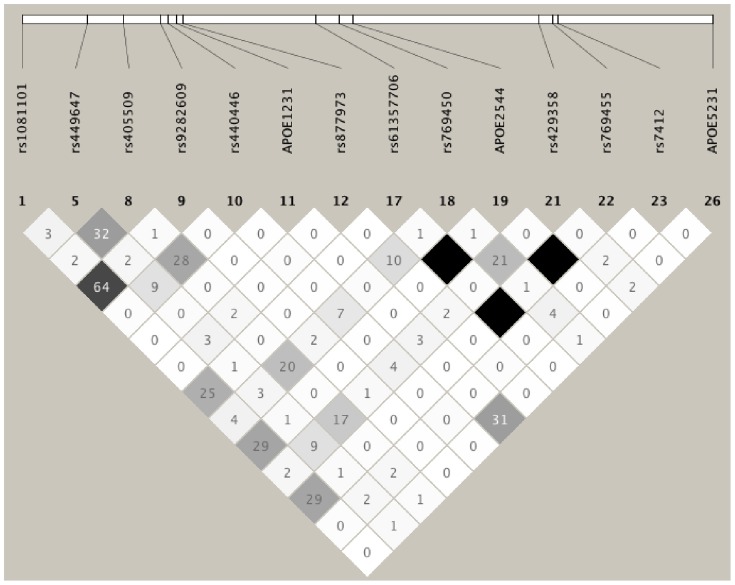
LD Plot of the genotyped variants with MAF>1% in African Blacks. The values in the cells are the pairwise degree of LD indicated by *r^2^*×100. *r* ^2^ = 0 is shown as white, 0<*r* ^2^<1 is shown in gray and *r* ^2^ = 1 is shown in black.

In African Blacks, 23 variants with high call rate and in compliance with HWE were included in the association analyses and their single-site association results are shown in **Table 2**. The LD plot of the genotyped variants with MAF>1% is shown in **Fig. 2** and the adjusted mean of the evaluated lipid traits among the genotype groups are presented in **Table S6 in S1 File**. As expected, the E*4 (rs429358) and E*2 (rs7412) SNPs were associated with LDL-C (β = 0.46; p = 0.0317 and β = −2.05; p = 5.35E-07, respectively). Four additional variants also showed association with LDL-C independent of E*2/E*4: *APOE*2269/rs61357706 in intron 2 (β = −2.23; p = 0.0034; FDR = 0.02), *APOE*2544/rs115299243 in intron 2 (β = −2.54; p = 0.0008; FDR = 0.008), *APO*E4036/rs769455(Arg163Cys) in exon 4 (β = −2.41; p = 0.0004; FDR = 0.008) and a novel association in 3′UTR, *APOE*4569 (β = 8.35; p = 0.024; FDR = 0.124). Two of these variants were also associated with TG *APO*E4036/rs769455 (Arg163Cys) (p = 0.0343; FDR = 0.199) and *APOE*2544/rs115299243 (p = 0.0378; FDR = 0.199). Two additional variants were also found to be associated with TG: *APO*E73/rs1081101 (p = 0.0115; FDR = 0.145), and *APOE*1279/rs877973 (p = 0.014; FDR = 0.145). One novel rare variant (*APOE*618) located in 5′ flanking region and observed in one individual was associated with extremely low HDL-C (13.5 mg/dl vs. 47.8 mg/dl; (β = −12.18; p =  0.001; FDR = 0.020); **Table S6 in S1 File**).

**Table 2:** Single-site association analysis with lipid traits in African Blacks.

	Variant					LDL-C^a^					ApoB^a^					TG^a^					HDL-C^a^				
								E*2/E*4 Adjusted Results					E*2/E*4 Adjusted Results					E*2/E*4 Adjusted Results					E2/E4 Adjusted Results		
Nucleotide position	Ch. Position	MAF	HWE-P	Location	RegulomeDB score	Beta	P	Adj. Beta	Adj. P	FDR	Beta	P	Adj. Beta	Adj. P	FDR	Beta	P	Adj. Beta	Adj. P	FDR	Beta	P	Adj. Beta	Adj. P	FDR
*APOE*73/rs1081101	44904820	0.0611	0.192	5′flanking	4	−0.62	0.111	−0.63	0.1079	0.268	0.65	0.5488	0.554	0.6126	0.774	0.038	**0.0093**	0.037	**0.0115**	**0.145**	0.30	0.4273	0.34	0.3860	0.737
*APOE*173	44904920	0.002	1	5′flanking	3a	−1.14	0.5979	−0.80	0.7083	0.816	3.20	0.5923	3.766	0.5279	0.759	0.037	0.7028	0.044	0.6520	0.856	−2.10	0.3206	−2.44	0.2558	0.672
*APOE*308/rs769445	44905055	0.0072	1	5′flanking	4	−0.37	0.7576	−0.35	0.7759	0.816	1.87	0.5514	1.993	0.5425	0.759	0.056	0.2065	0.038	0.4138	0.723	−1.06	0.3428	−1.17	0.3215	0.679
*APOE*560/rs449647	44905307	0.3663	0.348	5′flanking	5	0.58	**0.0031**	0.41	0.0590	0.237	0.92	0.0951	0.998	0.0958	0.503	−0.001	0.8482	0.003	0.7155	0.884	−0.18	0.3564	−0.17	0.4407	0.766
*APOE*618	44905365	0.0006	1	5′flanking	4	−2.85	0.4432	−2.86	0.4371	0.690	4.15	0.6876	3.953	0.7004	0.774	0.025	0.8560	0.019	0.8873	0.932	−12.25	**0.0008**	−12.18	**0.0010**	**0.020**
*APOE*624/rs769446	44905371	0.0077	1	5′flanking	3a	−2.86	**0.0214**	−0.97	0.4762	0.690	−4.19	0.2037	−3.816	0.2881	0.728	0.005	0.9096	0.039	0.4154	0.723	0.65	0.5687	0.31	0.8169	0.817
*APOE*832/rs405509	44905579	0.2561	0.039	5′flanking	1f	0.60	**0.0044**	0.39	0.0908	0.268	0.66	0.2611	0.506	0.4332	0.759	0.003	0.7457	0.005	0.5453	0.797	−0.19	0.3612	−0.12	0.6146	0.766
*APOE*1109/rs9282609	44905856	0.0415	0.035	Intron 1 (splice site)	4	0.05	0.9183	0.05	0.9155	0.915	1.38	0.2768	1.291	0.3120	0.728	0.023	0.1814	0.021	0.2238	0.587	0.61	0.1788	0.64	0.1679	0.609
*APOE*1163/rs440446	44905910	0.1004	0.681	intron1	4	0.44	0.1791	0.53	0.1148	0.268	2.30	**0.0109**	2.046	**0.0267**	0.380	0.023	0.0537	0.021	0.0879	0.369	−0.54	0.0946	−0.57	0.0944	0.609
*APOE*1231	44905978	0.0125	1	intron1	2b	−0.71	0.4139	−1.08	0.2266	0.476	−1.71	0.4748	−1.164	0.6400	0.774	−0.030	0.3441	−0.025	0.4478	0.723	−0.27	0.7550	−0.40	0.6640	0.766
*APOE*1279/rs877973	44906026	0.0597	0.336	intron1	4	−0.15	0.7208	−0.29	0.4927	0.690	−0.45	0.6847	−0.964	0.3962	0.759	−0.029	0.0513	−0.038	**0.0139**	**0.145**	0.51	0.1981	0.60	0.1427	0.609
*APOE*1539/rs184686013	44906286	0.0086	0.05	intron1	4	−1.03	0.2862	−0.81	0.4167	0.690	0.23	0.9330	0.470	0.8653	0.865	0.002	0.9546	−0.009	0.8082	0.893	1.35	0.1568	1.36	0.1739	0.609
*APOE*2072/rs189660912	44906819	0.0079	1	intron 2	4	−0.5	0.6433	−0.30	0.7774	0.816	0.70	0.8149	1.004	0.7374	0.774	0.009	0.8229	0.011	0.7900	0.893	−0.40	0.7066	−0.51	0.6391	0.766
*APOE*2269/rs61357706	44907016	0.0169	1	intron 2	5	−2.05	**0.0064**	−2.23	**0.0034**	**0.024**	−2.56	0.2201	−2.742	0.1965	0.688	0.040	0.1489	0.044	0.1234	0.432	0.77	0.2912	0.94	0.2127	0.638
*APOE*2440/rs769450	44907187	0.387	0.229	intron 2	5	0.20	0.3409	0.27	0.2659	0.508	−0.5	0.3725	−0.774	0.2323	0.697	−0.003	0.7396	−0.012	0.1730	0.519	−0.02	0.9394	0.13	0.6022	0.766
*APOE*2544/rs115299243	44907291	0.019	0.199	intron 2	5	−2.34	**0.0017**	−2.54	**0.0008**	**0.008**	−4.01	0.0524	−4.293	**0.0421**	0.380	0.061	**0.0470**	0.065	**0.0378**	**0.199**	−0.54	0.4827	−0.43	0.5866	0.766
*APOE*3673/rs769453	44908420	0.0066	1	intron 3	5	−0.37	0.7649	−0.36	0.7692	0.816	2.02	0.5386	1.990	0.5419	0.759	0.037	0.4242	0.037	0.4242	0.723	−1.10	0.3457	−1.17	0.3233	0.679
*APOE*3937/rs429358(E*4)	44908684	0.2656	0.518	exon 4 (Cys 130 Arg)	5	0.46	**0.0317**		-	-	0.05	0.9371				−0.008	0.3075				−0.14	0.5074			
*APOE*4036/rs769455	44908783	0.02	0.256	exon 4 (Arg 163 Cys)	5	−2.23	**0.0009**	−2.41	**0.0004**	**0.008**	−3.45	0.0664	−3.662	0.0543	0.380	0.056	**0.0372**	0.058	**0.0343**	**0.199**	−0.46	0.4827	−0.36	0.5940	0.766
*APOE*4075/rs7412(E*2)	44908822	0.0605	0.756	exon 4 (Arg 176 Cys)	5	−2.05	**5.35E-07**				−2.35	**0.0356**				−0.018	0.2376				0.75	0.0661			
*APOE*4569	44909316	0.0007	1	exon 4 (3′UTR)	5	8.87	**0.0173**	8.35	**0.0237**	**0.124**	14.25	0.1684	13.937	0.1748	0.688	0.073	0.5976	0.079	0.5689	0.797	5.29	0.1497	5.38	0.1480	0.609
*APOE*5223	44909970	0.0051	1	3′flanking	2b	−2.25	0.0874	−2.39	0.0676	0.237	−2.54	0.5166	−2.783	0.4756	0.759	0.006	0.9078	0.003	0.9486	0.949	0.39	0.763	0.46	0.7291	0.766
*APOE*5231	44909978	0.027	0.098	3′flanking	2b	−0.10	0.8587	−0.33	0.5736	0.753	−0.82	0.6036	−0.667	0.6784	0.774	−0.023	0.2836	−0.021	0.3504	0.723	−0.18	0.7645	−0.23	0.7160	0.766

Nucleotide position is according to the reference sequence (Accession # AF261279.1); Chr. Position: chromosomal position is according to NCBI dbSNP human Build 141. ^a^Box-Cox transformed variables. MAF: minor allele frequency. HWE-P: Hardy Weinberg equilibrium p-value. RegDB scores: RegulomeDB scores. LDL-C: low-density lipoprotein cholesterol; ApoB: Apolipoprotein B; TG: triglyceride; HDL-C: high-density lipoprotein cholesterol. **Bold** values represent significant p-values

### Gene-based association analysis

Gene-based tests including all *APOE* common and rare variants simultaneously within each ethnic group were performed (**Table 3** and **Table 4**). Gene-based association analysis showed significant associations (p<0.05) with TG, LDL-C and ApoB in NHWs and with LDL-C in African Blacks.

**Table 3:** Gene-based association analysis with lipid traits in NHWs.

Gene-based test based on all genotyped SNPs							
	Chr	Gene	nSNPs	Test	P-value	Best SNP	SNP p-value
HDL-C	19	APOE	13	19.99758	0.131	APOE1575/rs769448	0.019741
LDL-C	19	APOE	13	51.24064	**0.000391**	APOE4075/rs7412	1.84E-07
TG	19	APOE	13	28.89721	**0.02451**	APOE1163/rs440446	0.001807
ApoB	19	APOE	13	94.0465	**<1.00E-06**	APOE4075/rs7412	9.65E-13

Gene-based test based on genotyped SNPs with MAF ≥0.05							
	Chr	Gene	nSNPs	Test	P-value	Best SNP	SNP p-value
HDL-C	19	APOE	8	7.985102	0.417	APOE5361rs1081106	0.091454
LDL-C	19	APOE	8	48.96342	**0.000164**	APOE4075/rs7412	1.84E-07
TG	19	APOE	8	26.17339	**0.01205**	APOE1163/rs440446	0.001807
ApoB	19	APOE	8	86.75186	**1.00E-06**	APOE4075/rs7412	9.65E-13

nSNPs: represents the number of SNPs included in the analysis; two rare variants were excluded from the gene-based association analysis because of the missing phenotype data (ApoB data was available in a subset of the NHW sample); Test: represent the overall test statistic; P-value: the overall p-value; SNP p-value: p-value of the best SNPs contributed to the significance.

**Table 4:** Gene-based association analysis with lipid traits in African Blacks.

Gene-based test based on all genotyped SNPs							
	Chr	Gene	nSNPs	Test	P-value	Best SNP	SNP p-value
HDL-C	19	APOE	23	33.35805	0.122	APOE618	0.00079
LDL-C	19	APOE	23	97.16241	**8.00E-06**	APOE4075/rs7412	5.35E-07
TG	19	APOE	23	33.95383	0.124	APOE73/rs1081101	0.009268
APOB	19	APOE	23	32.04047	0.162	APOE1163/rs440446	0.010931

Gene-based test based on genotyped SNPs with MAF ≥0.05							
	Chr	Gene	nSNPs	Test	P-value	Best SNP	SNP p-value
HDL-C	19	APOE	8	10.58566	0.238	APOE4075/rs7412	0.066145
LDL-C	19	APOE	8	52.01841	**3.20E-05**	APOE4075/rs7412	5.35E-07
TG	19	APOE	8	16.97919	0.0552	APOE73/rs1081101	0.009268
APOB	19	APOE	8	16.2653	0.06542	APOE1163/rs440446	0.010931

nSNPs: represents the number of SNPs included in the analysis; Test: represent the overall test statistic; P-value: the overall p-value; SNP p-value: p-value of the best SNPs contributed to the significance.

### Haplotype-based association analysis

**Figure 3:**
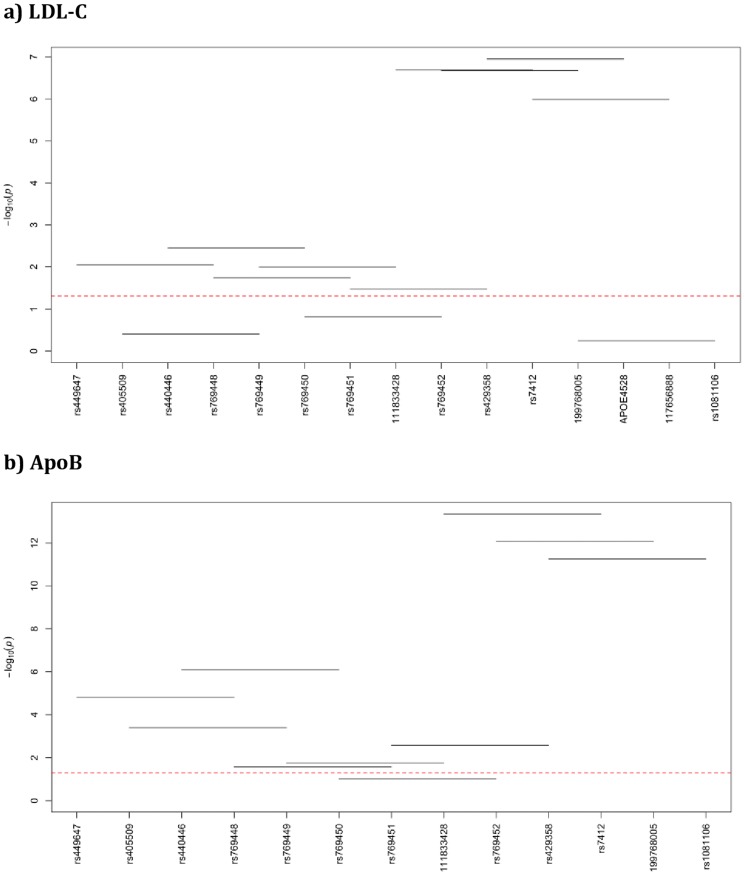
Haplotype analysis with lipid traits in NHWs. Haplotype windows for LDL-C (**a**), for ApoB (**b**), for HDL-C (**c**), and for TG (**d**). X-axis has the genotyped markers names and the Y-axis has the –log (global p-value), horizontal lines represent the 4-SNP windows, red-line represents the p-value threshold (p = 0.05) and everything below the threshold is considered non-significant and vice versa.

**Figure 4:**
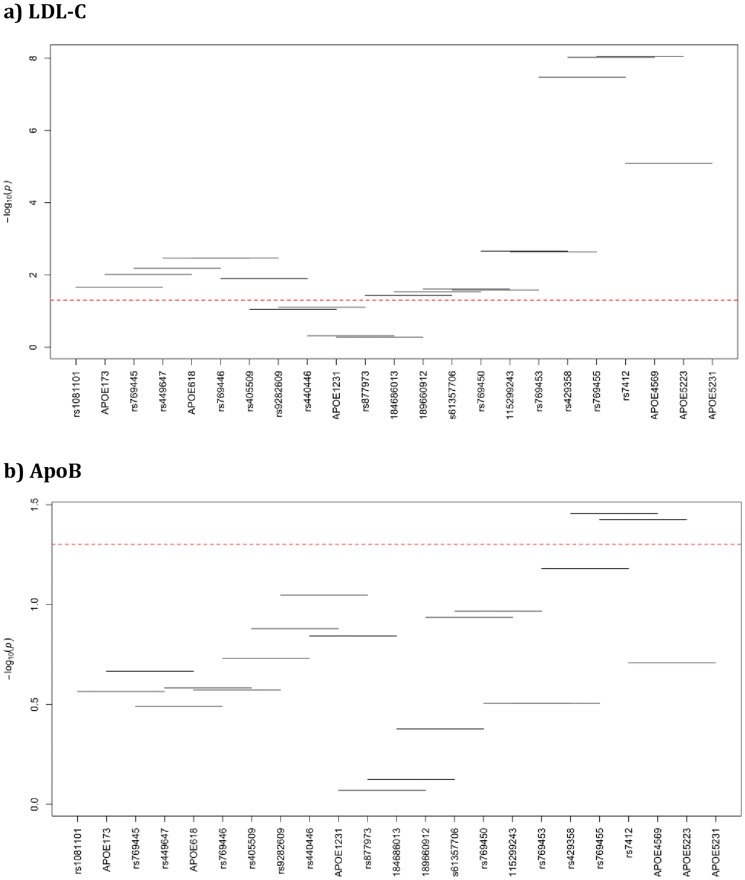
Haplotype analysis with lipid traits in African Blacks. Haplotype windows for LDL-C (**a**), for ApoB (**b**), for HDL-C (**c**), and for TG (**d**). X-axis has the genotyped markers name and the Y-axis has the –log (global p-value), horizontal lines represent the 4-SNP windows, red-line represents the p-value threshold (p = 0.05) and everything below the threshold is considered non-significant and vice versa.

The adjacent variants were evaluated as a group of four variants instead of relying on the effect of a single variant. The p-values for 4-SNP haplotype windows for each evaluated lipid trait are given in **Fig. 3**, and **Fig. 4** for NHWs and Blacks, respectively. For the haplotype-based association results please see the **Tables S7-S12 in S1 File** for NHWs and **Tables S13-S16 in S1 File** for Blacks.

In NHWs, the strongest haplotype associations were observed with ApoB followed by LDL-C. The region covered by five consecutive haplotype windows, including windows 7, 8, 9, 10 and 11 that harbor the variation in exon 4, showed the most significant global p-value with LDL-C (p-value ranges between 1.12E-07 and 0.0339), most likely due to the effect of E*2 (rs7412) and E*4 (rs429358) SNPs present in these windows. Additional four windows (1, 3, 4 and 5) showed nominally significant global p-value with LDL-C confirming the independent effect of *APOE*2440/rs769450 (p = 0.038). Similarly, the consecutive windows 7, 8, 9, and 10 that harbor variation in exon 4 showed significant haplotype global p-values with ApoB (p = 0.0027, 4.37E-14, 8.32E-13, and 5.47E-12) more likely due to the significant contribution of E*2 (rs7412) and E*4 (rs429358) on ApoB variation. Additionally the first five windows (windows 1, 2, 3, 4, and 5) showed significant global p-values (p = 1.57E-05, 0.0004, 8.05E-07, 0.0.0265, and 0.0176) more likely due to the effects of *APOE*832/rs405509 and *APOE*1998/rs769449 variants on ApoB Moreover, four windows (windows 1, 2, 3, and 5) showed independent evidence of association with TG (p = 0.0043, 0.0196, 0.0194, and 0.0344) likely to be mediated by the following three variants; *APOE*832/rs405509 (p = 0.003), *APOE*1163/rs440446 (p = 0.0018), and *APOE*2440/rs769450 (p = 0.0082) confirming their single-site effects. Only the last window, window 12 showed significant haplotype association with HDL-C (p = 0.0301), more likely due to the effect *APOE*4737/rs117656888.

In Blacks, the strongest haplotype associations were observed with LDL-C. Similar to NHWs, the last four windows (17, 18, 19, and 20), which include common polymorphisms in exon 4 showed the most significant p-values with LDL-C in African Blacks (p-values range between 8.86E-09 and 8.2E-06). Additional twelve windows showed significant effect on LDL-C (p-values range between 0.0022 and 0.036) including windows 1, 2, 3, 4, 5, 6, 11, 12, 13, 14, 15, and 16 and confirming the single-site effects of multiple variants (*APOE*560rs449647, *APOE*624/rs769446, *APOE*832/rs405509, *APOE*2269/rs61357706, and *APOE*2544/rs115299243) on LDL-C Unlike NHWs, only two windows (18, and 19) showed significant global p-value (0.035, and 0.038) with ApoB more likely due to the significant effect of E*2. Only the first window showed significant global p-value with TG (p = 0.035), most likely due to the effect of *APO*E73/rs1081101 as seen in the single-site analysis (p = 0.0093). Findings from haplotype-based association analyses confirm the single-site association results.

### Uncommon/Rare variants association analysis

Uncommon/rare variants association analysis was performed to examine the cumulative effect of uncommon/rare variants (MAF<5%) on lipid traits (HDL-C, LDL-C, and TG) using SKAT-O test. We found significant association with HDL-C in NHWs after including all 7 uncommon/rare variants in the analysis (p = 0.0061), and *APOE*1575/rs769448 with MAF = 0.021 contributed largely to this significance (**Table 5**) as it also showed the most significant association in single-site analysis (p = 0.0197). In Blacks, rare variants analysis (Table 6) showed significant association with LDL-C (p = 0.00018) and the significant association was driven by three variants with MAF between 0.017 and 0.020 (*APOE*2269/rs61357706, *APOE*2544/rs115299243, and *APOE*4036/rs769455), all of which showed significant association in single-site analysis (p range = 0.0009–0.0064).

**Table 5:** Rare/uncommon (MAF<5%) variants analysis with lipid traits in NHWs.

HDL-C						
MAF Threshold	MAF(≤0.01)		MAF(≤0.02)		MAF(≤ 0.05)	
Test	N.RV	P	N.RV	P	N.RV	P
SKAT-O	5	0.0922	6	0.1195	7	**0.0061**

LDL-C						
MAF Threshold	MAF(≤0.01)		MAF(≤0.02)		MAF(≤ 0.05)	
Test	N.RV	P	N.RV	P	N.RV	P
SKAT-O	5	0.2785	6	0.5605	7	0.7954

TG						
MAF Threshold	MAF(≤0.01)		MAF(≤0.02)		MAF(≤ 0.05)	
Test	N.RV	P	N.RV	P	N.RV	P
SKAT-O	5	0.167	6	0.3648	7	0.3369

SKAT-O: optimal sequencing Kernel association test, N.RV: number of rare variants, P: p-value.

**Table 6:** Rare/uncommon (MAF<5%) variants analysis with lipid traits in African Blacks.

HDL-C						
MAF Threshold	MAF(≤0.01)		MAF(≤0.02)		MAF(≤ 0.05)	
Test	N.RV	P	N.RV	P	N.RV	P
SKAT-O	9	0.5529	13	0.7315	15	0.7483

LDL-C						
MAF Threshold	MAF(≤0.01)		MAF(≤0.02)		MAF(≤ 0.05)	
Test	N.RV	P	N.RV	P	N.RV	P
SKAT-O	9	0.1147	13	**0.00018**	15	**0.00047**

TG						
MAF Threshold	MAF(≤0.01)		MAF(≤0.02)		MAF(≤ 0.05)	
Test	N.RV	P	N.RV	P	N.RV	P
SKAT-O	9	0.4503	13	0.1239	15	0.1323

SKAT-O: optimal sequencing Kernel association test, N.RV: number of rare variants, P: p-value.

### Functional annotation of the sequence variation

We used open-access database RegulomeDB (http://regulome.stanford.edu) to predict the potential implication of the identified genetic variation on the gene expression regulation. The RegulomeDB score of 1-5 is based on its strength of association with the gene regulation process; the lowest score represents the highest significant impact on regulation process (based on these features; expression quantitative trait loci (eQTL), transcription binding site or DNase hypersensitivity) while the highest score represents the least significant implication in regulation process. The RegulomeDB score for each variant is given in **Tables 1** **and** **2**. According to the RegulomeDB score, three variants in the 5′ flanking region (ApE173, ApE624/rs769446, and ApE832/rs405509), one intronic variant (ApE1231), and three variants in the 3′flanking region (ApE5223, ApE5231, and ApE5361/rs1081106) seem to affect gene expression as they have small scores (RegulomeDB score = 1–3). However, only two of these variants (*APOE* 624/rs769446 and *APOE*832/rs405509) had significant effect on LDL-C or ApoB or TG, and these two variants, *APOE*5223 and *APOE*5361/rs1081106, showed borderline effects on LDL-C and HDL-C, respectively. Although the remaining variants with strong regulatory effects (*APOE*173, *APOE*1231, and *APOE*5231) were not associated with lipid variation, they may yet have other biological consequences.

## Discussion

The role of common *APOE* genetic variation in affecting interindividual variation in plasma cholesterol, especially LDL-C, in the general population is well established. Less clear, however, is if *APOE* genetic variation has also an impact on other major lipid traits, like plasma HDL-C and TG. Recent lipid GWAS indicate that in addition to LDL-C, *APOE* common variants are also associated with HDL-C and TG levels [17]–[18]. Since common variants explain only ∼25–30% of the genetic variance of each major lipid trait [18], it has been hypothesized that uncommon low-frequency and rare variants in candidate genes may explain part of the missing heritability, as it has already been shown for some lipid genes [30]–[36]. Thus, deep resequencing of the *APOE* gene is warranted to identify both uncommon and common variants that might affect plasma lipid profile. The objective of this study was to evaluate the ‘common disease common variants’ (CDCV) and ‘common disease rare variants’ (CDRV) hypotheses by sequencing the entire *APOE* gene in selected individuals (n = 190) with extreme HDL-C levels from two ethnic groups in the variant discovery stage and then genotyping common tagSNPs and relevant uncommon/rare variants in the full datasets (NHWs = 623, and Blacks = 788) to evaluate their association with lipid traits. To our knowledge, this is the first population-based association study designed to evaluate the effect of the full spectrum of *APOE* genetic variation on major plasma lipid traits and ApoB levels. Previously, sequencing of the *APOE* gene has been reported in two different studies [38]–[39] and by the 1000 Genome project in order to characterize its genetic variation in unselected individuals without regards to lipid levels. Furthermore, most of the previous studies have only evaluated the influence of *APOE* coding and promoter variants on lipid traits [30]–[42].

By sequencing ∼5.5 kb of the *APOE* gene region, including all four exons, three introns, and ∼1 kb in each flanking region in selected individuals with extreme HDL-C levels in both population groups, we identified a total of 40 variants, including 10 novel variants not previously reported. As expected African Blacks tend to have more population-specific variants (21/31 = 68%) as compared to NHWs (9/19 = 47%). In NHWs, the proportion of common and uncommon variants was similar (56% vs. 44%), while in African Blacks more uncommon variants were observed than common ones (70% vs. 30%) (**Table S2 in S1 File**). We observed more subjects carrying group-specific uncommon variants in the high HDL-C group than in the low HDL-C group in NHWs (21% vs. 2%; p = 0.0037) and in African Blacks (15% vs. 6%; p = 0.316), although the difference in Blacks was not statistically significant. Likewise, the cumulative uncommon/rare variant analysis using SKAT-O also showed significant association with HDL-C in NHWs (p = 0.0061; **Table 5**).

The established association of the E*2 (rs7412) and E*4 (rs426538) SNPs with LDL-C and ApoB [15], [43]–[44] was confirmed in our study in which E*2 was associated with lowering effect on LDL-C (p = 1.84E-07 in NHWs, p = 5.35E-07 in Blacks), and ApoB (p = 9.65E-13 in NHWs, p = 0.0356 in Blacks), while E*4 was associated with elevating effect on LDL-C in both population groups (p = 0.0103 in NHWs; p = 0.0317 in Blacks) and elevating ApoB in NHWs (p = 0.0005). Although E*4 did not achieve the nominal significance with ApoB in Blacks, it showed similar trend of association. Moreover, we have identified 8 additional variants (4 in NHWs and 4 in Blacks) that were associated with LDL-C independent of the E*2 and E*4 SNPs. The 4 LDL-significant variants in NHWs include *APOE*832/rs405509,*APOE*1163/rs4405509, *APOE*4310/rs199768005(Val254Glu) and *APOE*2440/rs769450. While the first 3 variants are associated with lowering effect on LDL-C, the last variant was associated with elevating effect (see **Table 1**). Among the 4 LDL-associated variants in Blacks, 3 (*APOE*2269/rs61357706, *APOE*2544/rs115299243, and *APOE*4036/rs769455 (Arg163Cys) were associated with low LDL-C and this association is more likely mediated by *APOE*4036/rs769455 (Arg163Cys) that has previously been associated with type III hyperlipoproteinemia [50]–[51]. The fourth variant, *APOE*4569 (exon4), was associated with high LDL-C (see **Table 2**). While the 4 LDL-significant variants observed in Blacks were not detected in NHWs, 3 of the 4 LDL-significant variants in NHWs were observed in Blacks (*APOE*832/rs405509, *APOE*1163/rs4405509, and *APOE*2440/rs769450) and they also showed suggestive associations with LDL-C.

Of the above-mentioned 8 significant variants independent of the E*2 and E*4 SNPs only 3 (*APOE*832/rs405509, *APOE*1163/rs440446, and *APOE*4036/rs769455 (Arg163Cys)) have been examined previously in relation to lipid traits. *APOE*832/rs405509 located in the putative promoter region has previously been shown to be associated with LDL-related traits (LDL-C, TC, and ApoB) [16]–[17], [45], *APOE* gene expression [46], myocardial infarction risk [47], and premature CHD [48]. Our findings confirm the potentially important role of this variant in LDL metabolism by observing significant associations with LDL-C. *APOE*1163/rs440446 was earlier reported to be associated with CHD risk [49] and our current finding with its association with LDL-C validates this link given the relation between LDL-C levels and CHD risk. The non-synonymous variant *APOE*4036/rs769455 (Arg163Cys) has previously been reported to be associated with type III hyperlipoproteinemia [50]–[51] and is probably the main contributor to the significance signal of the two other closely linked variants (*APOE*2269/rs61357706 and *APOE*2544/rs115299243) with LDL-C.

In addition to the known contribution of *APOE* to LDL-C, we have found multiple associations of common and uncommon variants with TG and HDL-C. One NHW-specific uncommon variant (*APOE*1575/rs769448) was associated with elevating effect on HDL-C (p = 0.0223) and one rare Black-specific variant (*APOE*618) was associated with extremely low HDL-C, (p = 0.001), implying the significant contribution of *APOE* uncommon/rare variants on plasma HDL-C variation. To our knowledge, these are novel associations and need to be confirmed in independent studies. Based on their locations (intron 1, and 5′ flanking region, respectively), and RegulomeDB scores [4], they may be moderately involved in gene expression regulation. Nine variants showed significant association with TG, including five in NHWs: *APOE832*/rs405509 (p = 0.002), *APOE1163*/rs440446 (p = 0.0012), *APOE2440*/rs769450 (p = 0.0022), *APOE4310*/rs199768005 (p = 0.028), and *APOE4528*/rs374329439 (p = 0.0218) and four in Blacks: *APOE73*/rs1081101 (p = 0.0115), *APOE*1279/rs877973 (p = 0.014) *APOE2544*/rs115299243 (p = 0.038), and APOE4036/rs769455 (p = 0.0343). Four of these variants are uncommon, including two present only in NHWs (*APOE4310*/rs199768005/Val254Glu, and *APOE*4528/rs374329439) and two present only in African Blacks (*APOE*2544/rs115299243, and *APOE*4036/rs769455/Arg163Cys). Two of these population-specific variants involving non-synonymous changes (Arg163Cys, and Val254Glu) have previously been reported to be associated with type III hyperlipoproteinemia either in E*2-independent (rs769455/Arg163Cys) [50]–[51] or E*2-dependent (rs199768005/Val254Glu) [41] fashion. In our population-based samples while Arg163Cys was associated with higher TG levels, Val254Glu was associated with lower TG levels. The latter observation may not be surprising given that this variant was associated with hypertriglyceridemia only among E*2 carriers [41] and all our 5 subjects with this mutation in our study were non-E*2 carriers. This also implies that Val254Glu variant may be protective in the absence of E*2. In accordance with our observations, *APOE*832/rs405509 [17] has been found previously to be associated with VLDL as an indicator of TG variation and *APOE*1163/rs440446 [49] has previously been found to be associated with TG variation. To our knowledge, the remaining five TG associations observed in this study have not been reported previously and await confirmation in future studies.

In summary, this is the first comprehensive study that has evaluated the association of *APOE* common and rare variation with plasma lipid traits in two ethnic groups. In addition to the known association of common *APOE* variation with LDL-C, we have found that uncommon *APOE* variants also affect LDL-C levels. Our data also indicate the contribution of *APOE* genetic variation in affecting HDL-C and TG levels in the general population. Strengths of our study include the use of two extreme lipid groups for resequencing from two ethnic groups and then genotyping of the entire sample sets for genotype-phenotype association analyses. Limitations of our study include the use of relatively small sample sizes for resequencing. Many of our significant findings with uncommon/rare variants should be considered provisional until replicated in independent and large data sets.

## Supporting Information

S1 File **Table S1.** Demographic characteristics of the resequencing samples; **Table S2.** *APOE* sequencing variants identified in 95 NHWs and 95 African Blacks (n = 95); **Table S3.** Distribution of the sequence variants in the extreme HDL-C groups in NHWs (n = 95); **Table S4.** Distribution of the sequence variants in the extreme HDL-C groups in African Blacks (n = 95); **Table S5.** Single-site association analysis in NHWs (n = 623); **Table S6.** Single-site association analysis in African Blacks (n = 788); **Table S7.** 4-SNPs window haplotype-based association results for LDL-C, TG and HDL-C in NHWs (n = 623); **Table S8.** 4-SNPs window haplotype-based association results for ApoB in NHWs (n = 623); **Table S9.** Haplotype-based association summary of significant windows with LDL-C in NHWs (n = 623); **Table S10.** Haplotype-based association summary of significant windows with ApoB in NHWs (n = 623); **Table S11.** Haplotype-based association summary of significant windows with TG in NHWs (n = 623); **Table S12.** Haplotype-based association summary of significant windows with HDL-C in NHWs (n = 623); **Table S13.** 4-SNPs window haplotype-based association results for lipid traits in African Blacks (n = 788); **Table S14.** Haplotype-based association summary of significant windows with LDL-C in African Blacks (n = 788); **Table S15.** Haplotype-based association summary of significant windows with ApoB in African Blacks (n = 788); **Table S16.** Haplotype-based association summary of significant windows with TG in African Blacks (n = 788).
(DOCX)
