# Supplementary material for: Comprehensive Evaluation of the Association of APOE Genetic Variation with Plasma Lipoprotein Traits in U.S. Whites and African Blacks
Source: PLoS One. 2014 Dec 12;9(12):e114618. doi: 10.1371/journal.pone.0114618 (PMC4264772; doi:10.1371/journal.pone.0114618)
Supplement: S1 File — Table S1. Demographic characteristics of the resequencing samples; Table S2. APOE sequencing variants identified in 95 NHWs and 95 African Blacks (n = 95); Table S3. Distribution of the sequence variants in the extreme HDL-C groups in NHWs (n = 95); Table S4. Distribution of the sequence variants in the extreme HDL-C groups in African Blacks (n = 95); Table S5. Single-site association analysis in NHWs (n = 623); Table S6. Single-site association analysis in African Blacks (n = 788); Table S7. 4-SNPs window haplotype-based association results for LDL-C, TG and HDL-C in NHWs (n = 623); Table S8. 4-SNPs window haplotype-based association results for ApoB in NHWs (n = 623); Table S9. Haplotype-based association summary of significant windows with LDL-C in NHWs (n = 623); Table S10. Haplotype-based association summary of significant windows with ApoB in NHWs (n = 623); Table S11. Haplotype-based association summary of significant windows with TG in NHWs (n = 623); Table S12. Haplotype-based association summary of significant windows with HDL-C in NHWs (n = 623); Table S13. 4-SNPs window haplotype-based association results for lipid traits in African Blacks (n = 788); Table S14. Haplotype-based association summary of significant windows with LDL-C in African Blacks (n = 788); Table S15. Haplotype-based association summary of significant windows with ApoB in African Blacks (n = 788); Table S16. Haplotype-based association summary of significant windows with TG in African Blacks (n = 788). (DOCX) [file pone.0114618.s001.docx]

SUPPLEMENTARY TABLES AND FIGURES

Comprehensive evaluation of the association of APOE genetic variation with plasma lipoprotein traits in U.S. Whites and African Blacks

Zaheda H. Radwan^1^, Xingbin Wang^1^, Fahad Waqar^1^, Dilek Pirim^1^, Vipavee Niemsiri^1^, John E. Hokanson^2^, Richard F. Hamman^2^, Clareann H. Bunker^3^, M. Michael Barmada^1^, F. Yesim Demirci^1^, M. Ilyas Kamboh^1^

^1^Department of Human Genetics, Graduate School of Public Health, University of Pittsburgh, Pittsburgh, PA

^2^Department of Epidemiology, Colorado School of Public Health, University of Colorado Denver, Aurora, CO

^3^Department of Epidemiology, Graduate School of Public Health, University Pittsburgh, Pittsburgh, PA

**Address for correspondence:** M. Ilyas Kamboh, Ph.D, Department of Human Genetics, Graduate School of Public Health, University of Pittsburgh, 130 DeSoto Street, Pittsburgh, PA 15261, USA. e-mail: [kamboh@pitt.edu](mailto:kamboh@pitt.edu)

Table S1. Demographic characteristics of the resequencing samples

|  | **NHWs (n=95)** | | | **African Blacks (n=95)** | | |
| --- | --- | --- | --- | --- | --- | --- |
|  | **High HDL-C (n=47)** | **Low HDL-C (n=48)** | **P-value** | **High HDL-C (n=48)** | **Low HDL-C (n=47)** | **P-value** |
| **Sex (M/F)** | 24/23 | 24/24 | 1.0 | 24/24 | 23/24 | 1.0 |
| **Age (Yrs)** | 55.45 ± 9.8 | 53.03 ± 10.54 | 0.25 | 41.29 ± 8.72 | 40.87 ± 7.12 | 0.8 |
| **BMI (kg/m^2)^** | 23.17 ± 3.17 | 27.35 ± 3.90 | <0.001 | 22.06 ± 4.70 | 23.91 ± 5.51 | 0.08 |
| **TOTAL-C (mg/dl)** | 227.34 ± 51.76 | 208.81 ± 44.65 | 0.07 | 201 ± 39.68 | 141.68 ± 31.03 | <0.001 |
| **LDL-C (mg/dl)** | 126.84 ± 46.95 | 125.54± 54.97 | 0.90 | 112.55 ± 39.75 | 95.04 ± 28.28 | 0.02 |
| **TG (mg/dl)** | 114.09 ± 60.88 | 240.21 ± 153.22 | <0.001 | 61.98 ± 19.85 | 95.79 ± 73.21 | 0.004 |
| **HDL-C (mg/dl)** | 77.68 ± 13.32 | 31.81 ± 4.37 | <0.001 | 76.05 ± 7.53 | 25.51 ± 5.66 | <0.001 |
| **ApoB (mg/dl)** | 87.88 ± 25.49 | 89.61 ± 25.18 | 0.80 | 66.00 ± 22.20 | 69.64 ± 21.46 | 0.40 |
| *P-values were calculated based on the original values by using t-test. No covariates were included. The correlation values between lipid traits are as follows: between LDL-C and HDL-C (0.02 in NHWs, 0.11 in Blacks); between LDL-C and ApoB (0.46 in NHWs, 0.58 in Blacks); between HDL-C and TG (-0.33 in NHWs, -0.21 in Blacks); between TG and ApoB (0.07 in NHWs and 0.17 in Blacks).* | | | | | | |

Table S2. *APOE* sequencing variants identified in 95 NHWs and 95 African Blacks (n=95)

| **Nucleotide Position** | **Alleles** | **Chr. Position** | **NCBI refSNP ID** | **Location** | **Amino Acid Change** | **NHWs** | | | **African Blacks** | | | **RegulomeDB score** |
| --- | --- | --- | --- | --- | --- | --- | --- | --- | --- | --- | --- | --- |
|  |  |  |  |  |  | **MAF** | **HWE-P** | **%Call rate** | **MAF** | **HWE-P** | **%Call rate** |  |
| APOE73 | C>T | 44904820 | rs1081101 | 5'flanking |  | *** | *** | *** | 0.074 | 1.000 | 98.9 | 4 |
| APOE173 | A>G | 44904920 |  | 5'flanking |  | *** | *** | *** | 0.005 | 1.000 | 100 | 3a |
| APOE308 | C>T | 44905055 | rs769445 | 5'flanking |  | *** | *** | *** | 0.005 | 1.000 | 100 | 4 |
| APOE471 | A>G | 44905218 | rs439382 | 5'flanking |  | *** | *** | *** | 0.132 | 1.000 | 100 | 7 |
| APOE494 | C>T | 44905241 |  | 5'flanking |  | *** | *** | *** | 0.005 | 1.000 | 100 | 7 |
| APOE526 | T>C | 44905273 |  | 5'flanking |  | *** | *** | *** | 0.005 | 1.000 | 100 | 5 |
| APOE560 | A>T | 44905307 | rs449647 | 5'flanking |  | 0.126 | 0.3791 | 100 | 0.395 | 0.169 | 100 | 5 |
| APOE618 | G>C | 44905365 |  | 5'flanking |  | *** | *** | *** | 0.005 | 1.000 | 100 | 4 |
| APOE624 | T>C | 44905371 | rs769446 | 5'flanking |  | 0.075 | 1.000 | 97.9 | 0.005 | 1.000 | 100 | 3a |
| APOE832 | G>T | 44905579 | rs405509 | 5'flanking |  | 0.484 | 1.000 | 100 | 0.261 | 0.336 | 98.9 | 1f |
| APOE1109 | C>T | 44905856 | rs9282609 | Intron 1 (splice site) |  | *** | *** | *** | 0.053 | 1.000 | 98.9 | 4 |
| APOE1163 | G>C | 44905910 | rs440446 | intron1 |  | 0.358 | 0.108 | 100 | 0.124 | 0.821 | 97.9 | 4 |
| APOE1231 | G>A | 44905978 |  | Intron 1 |  | *** | *** | *** | 0.011 | 1.000 | 98.9 | 2b |
| APOE1279 | C>A | 44906026 | rs877973 | Intron 1 |  | *** | *** | *** | 0.048 | 1.000 | 98.9 | 4 |
| APOE1539 | A>G | 44906286 | rs184686013 | Intron 1 |  | *** | *** | *** | 0.011 | 1.000 | 100 | 4 |
| APOE1575 | C>T | 44906322 | rs769448 | Intron 1 |  | 0.021 | 1.000 | 100 | *** | *** | *** | 4 |
| APOE1591 | G>T | 44906338 | rs147236548 | Intron 1 |  | *** | *** | *** | 0.016 | 1.000 | 100 | 2b |
| APOE1998 | G>A | 44906745 | rs769449 | intron 2 |  | 0.126 | 1.000 | 100 | *** | *** | *** | 4 |
| APOE2072 | G>A | 44906819 | rs189660912 | intron 2 |  | *** | *** | *** | 0.016 | 1.000 | 100 | 4 |
| APOE2269 | G>A | 44907016 | rs61357706 | intron 2 |  | *** | *** | *** | 0.016 | 1.000 | 98.9 | 5 |
| APOE2294 | C>T | 44907041 |  | intron 2 |  | 0.005 | 1.000 | 100 | *** | *** | *** | 5 |
| APOE2440 | G>A | 44907187 | rs769450 | intron 2 |  | 0.404 | 0.855 | 100 | 0.394 | 0.977 | 98.9 | 5 |
| APOE2544 | A>G | 44907291 | rs115299243 | intron 2 |  | *** | *** | *** | 0.016 | 1.000 | 98.9 | 5 |
| APOE2576 | G>A | 44907323 |  | intron 2 |  | *** | *** | *** | 0.005 | 1.000 | 100 | 7 |
| APOE2907 | T>G | 44907654 | rs769451 | intron 2 |  | 0.005 | 1.000 | 100 | *** | *** | *** | 5 |
| APOE3038 | G>A | 44907785 | rs111833428 | exon 3 | Ala 23 Ala | 0.005 | 1.000 | 100 | *** | *** | *** | 5 |
| APOE3673 | C>G | 44908420 | rs769453 | intron 3 |  | *** | *** | *** | 0.005 | 1.000 | 100 | 5 |
| APOE3937 | T>C | 44908684 | rs429358 (E*4) | exon 4 | Cys 130 Arg | 0.168 | 1.000 | 100 | 0.237 | 0.21 | 100 | 5 |
| APOE4036 | C>T | 44908783 | rs769455 | exon 4 | Arg 163 Cys | *** | *** | *** | 0.016 | 1.000 | 96.8 | 5 |
| APOE4075 | C>T | 44908822 | rs7412(E*2) | exon 4 | Arg 176 Cys | 0.063 | 1.000 | 100 | 0.042 | 1.000 | 100 | 5 |
| APOE4310 | T>A | 44909057 | rs199768005 | exon 4 | Val 254 Glu | 0.005 | 1.000 | 100 | *** | *** | *** | 5 |
| APOE4528 | C>T | 44909275 | rs374329439 | exon 4 (3' UTR) |  | 0.005 | 1.000 | 100 | *** | *** | *** | 5 |
| APOE4569 | G>T | 44909316 |  | exon 4 (3' UTR) |  | *** | *** | *** | 0.005 | 1.000 | 100 | 5 |
| APOE4737 | C>G | 44909484 | rs117656888 | 3'flanking |  | 0.011 | 1.000 | 100 | *** | *** | *** | 5 |
| APOE4951 | A>C | 44909698 | rs1081105 | 3'flanking |  | 0.042 | 1.000 | 100 | 0.042 | 1.000 | 100 | 5 |
| APOE5223 | G>C | 44909970 |  | 3'flanking |  | *** | *** | *** | 0.005 | 1.000 | 100 | 2b |
| APOE5229 | G>T | 44909976 | rs80125357 | 3'flanking |  | 0.064 | 1.000 | 98.9 | 0.059 | 1.000 | 98.9 | 2b |
| APOE5229_5230 | indelG | 44909977 | rs55729972 | 3'flanking |  | …… | …… | …… | …… | …… | …… | 2b |
| APOE5231 | T>G | 44909978 |  | 3'flanking |  | *** | *** | *** | 0.032 | 1.000 | 100 | 2b |
| APOE5361 | T>C | 44910109 | rs1081106 | 3'flanking |  | 0.105 | 1.000 | 100 | *** | *** | *** | 3a |

Nucleotide position is according to the reference sequence (Accession # AF261279.1); Chr. Position: chromosomal position is according to NCBI dbSNP human Build 141; (***) Unavailable data for population-specific variants, (……) not analyzed indel; HWE-P (Hardy Weinberg equilibrium p-value).

Table S3. Distribution of the sequence variants in the extreme HDL-C groups in NHWs (n=95)

| **Common variants (MAF≥5%)** | | | | | | | | | |
| --- | --- | --- | --- | --- | --- | --- | --- | --- | --- |
| **Nucleotide Position** | **Alleles** | **Chr. Position** | **NCBI refSNP ID** | **Location** | **a.a. change** | **RegulomeDB score** | **MAF** | **MAF in High HDL-C (n=47)** | **MAF in Low HDL-C (n=48)** |
| APOE560 | A>T | 44905307 | rs449647 | 5'flanking |  | 5 | 0.126 | 0.085 | 0.167 |
| APOE624 | T>C | 44905371 | rs769446 | 5'flanking |  | 3a | 0.075 | 0.111 | 0.042 |
| APOE832 | G>T | 44905579 | rs405509 | 5'flanking |  | 1f | 0.484 | 0.479 | 0.49 |
| APOE1163 | G>C | 44905910 | rs440446 | intron1 |  | 4 | 0.358 | 0.34 | 0.375 |
| APOE1998 | G>A | 44906745 | rs769449 | intron 2 |  | 4 | 0.126 | 0.128 | 0.125 |
| APOE2440 | G>A | 44907187 | rs769450 | intron 2 |  | 5 | 0.4 | 0.404 | 0.396 |
| APOE3937 | T>C | 44908684 | rs429358 | exon 4 | Cys 130 Arg | 5 | 0.168 | 0.16 | 0.177 |
| APOE4075 | C>T | 44908822 | rs7412 | exon 4 | Arg 176 Cys | 5 | 0.063 | 0.074 | 0.052 |
| APOE5229 | G>T | 44909976 | rs80125357 | 3'flanking |  | 2b | 0.064 | 0.076 | 0.052 |
| APOE5361 | T>C | 44910109 | rs1081106 | 3'flanking |  | 3a | 0.105 | 0.117 | 0.094 |
| **Uncommon/rare variants with (MAF<5%)** | | | | | | | | | |
| **Nucleotide Position** | **Alleles** | **Chr. Position** | **NCBI refSNP ID** | **Location** | **a.a. change** | **RegulomeDB score** | **MAF** | **MAF in High HDL-C (n=47)** | **MAF in Low HDL-C (n=48)** |
| APOE1575 | C>T | 44906322 | rs769448 | intron1 |  | 4 | 0.021 | 0.043 | 0 |
| APOE2294 | C>T | 44907041 |  | intron 2 |  | 5 | 0.005 | 0.011 | 0 |
| APOE2907 | T>G | 44907654 | rs769451 | intron 2 |  | 5 | 0.005 | 0 | 0.01 |
| APOE3038 | G>A | 44907785 | rs111833428 | exon 3 | Ala 23 Ala | 5 | 0.005 | 0.011 | 0 |
| APOE4310 | T>A | 44909057 | rs199768005 | exon 4 | Val 254 Glu | 5 | 0.005 | 0.011 | 0 |
| APOE4528 | C>T | 44909275 | rs374329439 | exon 4 (3' UTR) |  | 5 | 0.005 | 0.011 | 0 |
| APOE4737 | C>G | 44909484 | rs117656888 | 3'flanking |  | 5 | 0.011 | 0.021 | 0 |
| APOE4951 | A>C | 44909698 | rs1081105 | 3'flanking |  | 5 | 0.042 | 0.032 | 0.052 |

Nucleotide position is according to the reference sequence (Accession # AF261279.1); Chr. Position: chromosomal position is according to NCBI dbSNP human Build 141; Alleles change is in form of reference allele to mutant allele change; MAF is minor allele frequency in the sequencing subset (95 NHWs).

Table S4. Distribution of the sequence variants in the extreme HDL-C groups in African Blacks (n=95)

| **Common variants (MAF≥5%)** | | | | | | | | | |
| --- | --- | --- | --- | --- | --- | --- | --- | --- | --- |
| **Nucleotide Position** | **Alleles** | **Chr. Position** | **NCBI RefSNP ID** | **Location** | **a.a change** | **RegulomeDB score** | **MAF** | **MAF in High HDL-C** | **MAF in Low HDL-C** |
|  |  |  |  |  |  |  |  | **(n=48)** | **(n=47)** |
| APOE73 | C>T | 44904820 | rs1081101 | 5'flanking |  | 4 | 0.074 | 0.062 | 0.087 |
| APOE471 | A>G | 44905218 | rs439382 | 5'flanking |  | 7 | 0.132 | 0.125 | 0.138 |
| APOE560 | A>T | 44905307 | rs449647 | 5'flanking |  | 5 | 0.395 | 0.385 | 0.404 |
| APOE832 | G>T | 44905579 | rs405509 | 5'flanking |  | 1f | 0.261 | 0.26 | 0.261 |
| APOE1109 | C>T | 44905856 | rs9282609 | splice site |  | 4 | 0.053 | 0.052 | 0.054 |
| APOE1163 | G>C | 44905910 | rs440446 | intron1 |  | 4 | 0.124 | 0.115 | 0.133 |
| APOE2440 | G>A | 44907187 | rs769450 | intron 2 |  | 5 | 0.394 | 0.383 | 0.404 |
| APOE3937 | T>C | 44908684 | rs429358 | exon 4 | Cys 130 Arg | 5 | 0.237 | 0.24 | 0.234 |
| APOE5229 | G>T | 44909976 | rs80125357 | 3'flanking |  | 2b | 0.059 | 0.074 | 0.043 |
| **Uncommon/rare variants (MAF<5%)** | | | | | | | | | |
| **Nucleotide Position** | **Alleles** | **Chr. Position** | **NCBI RefSNP ID** | **Location** | **a.a change** | **RegulomeDB score** | **MAF** | **MAF in High HDL-C** | **MAF in Low HDL-C** |
|  |  |  |  |  |  |  |  | **(n=48)** | **(n=47)** |
| APOE173 | A>G | 44904920 |  | 5'flanking |  | 3a | 0.005 | 0 | 0.011 |
| APOE308 | C>T | 44905055 | rs769445 | 5'flanking |  | 4 | 0.005 | 0 | 0.011 |
| APOE494 | C>T | 44905241 |  | 5'flanking |  | 7 | 0.005 | 0.01 | 0 |
| APOE526 | T>C | 44905273 |  | 5'flanking |  | 5 | 0.005 | 0.01 | 0 |
| APOE618 | G>C | 44905365 |  | 5'flanking |  | 4 | 0.005 | 0 | 0.011 |
| APOE624 | T>C | 44905371 | rs769446 | 5'flanking |  | 3a | 0.005 | 0.01 | 0 |
| APOE1231 | G>A | 44905978 |  | intron1 |  | 2b | 0.011 | 0.01 | 0.011 |
| APOE1279 | C>A | 44906026 | rs877973 | intron1 |  | 4 | 0.048 | 0.062 | 0.033 |
| APOE1539 | A>G | 44906286 | rs184686013 | intron1 |  | 4 | 0.011 | 0.021 | 0 |
| APOE1591 | G>T | 44906338 | rs147236548 | intron1 |  | 2b | 0.016 | 0.021 | 0.011 |
| APOE2072 | G>A | 44906819 | rs189660912 | intron 2 |  | 4 | 0.016 | 0.021 | 0.011 |
| APOE2269 | G>A | 44907016 | rs61357706 | intron 2 |  | 5 | 0.016 | 0.01 | 0.022 |
| APOE2544 | A>G | 44907291 | rs115299243 | intron 2 |  | 5 | 0.016 | 0.01 | 0.022 |
| APOE2576 | G>A | 44907323 |  | intron 2 |  | 7 | 0.005 | 0.01 | 0 |
| APOE3673 | C>G | 44908420 | rs769453 | intron 3 |  | 5 | 0.005 | 0 | 0.011 |
| APOE4036 | C>T | 44908783 | rs769455 | exon 4 | Arg 163 Cys | 5 | 0.016 | 0.011 | 0.022 |
| APOE4075 | C>T | 44908822 | rs7412 | exon 4 | Arg 176 Cys | 5 | 0.042 | 0.062 | 0.021 |
| APOE4569 | G>T | 44909316 |  | exon 4 (3'UTR) |  | 5 | 0.005 | 0.01 | 0 |
| APOE4951 | A>C | 44909698 | rs1081105 | 3'flanking |  | 5 | 0.042 | 0.042 | 0.043 |
| APOE5223 | G>C | 44909970 |  | 3'flanking |  | 2b | 0.005 | 0 | 0.011 |
| APOE5231 | T>G | 44909978 |  | 3'flanking |  | 2b | 0.032 | 0.042 | 0.021 |

Nucleotide position is according to the reference sequence (Accession # AF261279.1); Chr. Position: chromosomal position is according to NCBI dbSNP human Build 141, Alleles change is in form of reference allele to mutant allele change, MAF is minor allele frequency in the sequencing subset (95 African Blacks).

Table S5. Single-site association analysis in NHWs (n=623)

| **LDL-C** | | | | | | | | | | |
| --- | --- | --- | --- | --- | --- | --- | --- | --- | --- | --- |
| **Variant name** | **Chr. Position** | **Location** | **RegulomeDB score** | **MAF** | **Genotype** | **GT Count** | **GT_AdjMean** | **GT_SD** | **Beta** | **P-value** |
| APOE560/rs449647 | 44905307 | 5' flanking | 5 | 0.161 | AA/AT/TT | 433/176/12 | 139.19/132.29/124.15 | 38.8/45.3/36.3 | -7.12 | **0.0247** |
| APOE832/rs405509 | 44905579 | 5' flanking | 1f | 0.4775 | GG/GT/TT | 170/310/142 | 135.68/138.13/136.32 | 40.3/42.7/37.3 | 0.42 | 0.8542 |
| APOE1163/rs440446 | 44905910 | Intron 1 | 4 | 0.3604 | CC/GC/GG | 76/297/250 | 130.59/137.86/137.91 | 37.8/40.7/41.7 | -2.59 | 0.281 |
| APOE1575/rs769448 | 44906322 | Intron 1 | 4 | 0.021 | CC/CT/TT | 595/24/1 | 136.96/137.36/142.42 | 41.3/29.6/NA | 0.75 | 0.9232 |
| APOE1998/rs769449 | 44906745 | intron 2 | 4 | 0.1165 | AA/AG/GG | 6/132/480 | 160.24/141.21/135.47 | 19.9/41.4/40.7 | 6.95 | 0.0551 |
| APOE2440/rs769450 | 44907187 | intron 2 | 5 | 0.4015 | AA/GA/GG | 95/307/217 | 144.97/136.5/133.97 | 40.1/40.9/40.9 | 4.89 | **0.0378** |
| APOE2907/rs769451 | 44907654 | intron 2 | 5 | 0.0112 | GT/TT | 14/609 | 137.43/136.98 | 36.6/40.9 | 0.45 | 0.9668 |
| APOE3038/rs111833428 | 44907785 | exon 3 (Ala 23 Ala) | 5 | 0.0016 | AG/GG | 2/616 | 164.05/136.78 | 57.3/40.9 | 27.34 | 0.3354 |
| APOE3106/rs769452 | 44907853 | exon 3 (Leu46Pro) | 5 | 0.0008 | TC/TT | 1/620 | 154.92/136.93 | NA/40.9 | 18.03 | 0.6522 |
| APOE3937/rs429358 | 44908684 | exon 4 (Cys 130 Arg) | 5 | 0.1525 | CC/CT/TT | 14/159/440 | 153.24/142.39/134.84 | 23.3/39.4/41.2 | 8.10 | **0.0103** |
| APOE4075/rs7412 | 44908822 | exon 4 (Arg 176 Cys) | 5 | 0.0806 | CC/TC/TT | 523/94/3 | 140.26/120.22/73.75 | 39.2/44.6/37.0 | -21.84 | **1.84E-07** |
| APOE4310/rs199768005 | 44909057 | exon 4 (Val 254 Glu) | 5 | 0.004 | TA/TT | 5/617 | 105/137.26 | 35.7/40.8 | -32.56 | 0.0705 |
| APOE4528/rs374329439 | 44909275 | exon 4 (3' UTR) | 5 | 0.0008 | CC/CT | 622/1 | 137.01/124.75 | 40.8/NA | -12.3 | 0.7585 |
| APOE4737/rs117656888 | 44909484 | 3'flanking | 5 | 0.0081 | CC/GC | 610/10 | 136.68/150.24 | 40.7/52.3 | 13.58 | 0.2867 |
| APOE5361/rs1081106 | 44910109 | 3'flanking | 3a | 0.0852 | CC/TC/TT | 4/98/520 | 163.88/138.72/136.47 | 28.8/37.4/41.5 | 4.00 | 0.3272 |
| **ApoB^a^** | | | | | | | | | | |
| **Variant name** | **Chr. Position** | **Location** | **RegulomeDB score** | **MAF** | **Genotype** | **GT Count** | **GT_AdjMean** | **GT_SD** | **Beta** | **P-value** |
| APOE560/rs449647 | 44905307 | 5' flanking | 5 | 0.161 | AA/AT/TT | 306/119/7 | 88.92/84.42/90.53 | 23.4/24.7/13.2 | -0.95 | 0.129 |
| APOE832/rs405509 | 44905579 | 5' flanking | 1f | 0.4775 | GG/GT/TT | 115/217/102 | 81.67/89.51/91.58 | 26.1/22.6/22.9 | 1.46 | **0.0009** |
| APOE1163/rs440446 | 44905910 | Intron 1 | 4 | 0.3604 | CC/GC/GG | 51/206/177 | 88.2/89.54/85.95 | 23.0/23.5/24.8 | 0.56 | 0.2319 |
| APOE1575/rs769448 | 44906322 | Intron 1 | 4 | 0.021 | CC/CT/TT | 413/17/1 | 87.88/90.73/93.77 | 24.3/17.4/NA | 0.94 | 0.5204 |
| APOE1998/rs769449 | 44906745 | intron 2 | 4 | 0.1165 | AA/AG/GG | 4/103/324 | 93.32/93.83/85.99 | 23.2/22.4/24.1 | 2.03 | **0.0030** |
| APOE2440/rs769450 | 44907187 | intron 2 | 5 | 0.4015 | AA/GA/GG | 63/217/153 | 89.59/87.42/87.95 | 24.1/23.9/24.1 | 0.15 | 0.7422 |
| APOE2907/rs769451 | 44907654 | intron 2 | 5 | 0.0112 | GT/TT | 9/425 | 87.57/87.92 | 32.9/23.8 | -0.22 | 0.9196 |
| APOE3038/rs111833428 | 44907785 | exon 3 (Ala 23 Ala) | 5 | 0.0016 | AG/GG | 1/431 | 140.55/87.71 | NA/23.8 | 13.81 | **0.0342** |
| APOE3106/rs769452 | 44907853 | exon 3 (Leu46Pro) | 5 | 0.0008 | TC/TT | 1/432 | 121.87/87.93 | NA/23.9 | 9.15 | 0.1602 |
| APOE3937/rs429358 | 44908684 | exon 4 (Cys 130 Arg) | 5 | 0.1525 | CC/CT/TT | 9/121/296 | 90.86/94.72/85.3 | 17.2/22.9/24.1 | 2.14 | **0.0005** |
| APOE4075/rs7412 | 44908822 | exon 4 (Arg 176 Cys) | 5 | 0.0806 | CC/TC/TT | 365/65/3 | 90.92/73.55/32.32 | 23.0/21.1/17.1 | -5.60 | **9.65E-13** |
| APOE4310/rs199768005 | 44909057 | exon 4 (Val 254 Glu) | 5 | 0.004 | TA/TT | 2/431 | 92.47/87.91 | 45.3/23.9 | 1.05 | 0.822 |
| APOE5361/rs1081106 | 44910109 | 3'flanking | 3a | 0.0852 | CC/TC/TT | 3/59/372 | 107.81/86.35/88.01 | 28.0/23.3/24.0 | 0.12 | 0.8813 |
| **TG^a^** | | | | | | | | | | |
| **Variant name** | **Chr. Position** | **Location** | **RegulomeDB score** | **MAF** | **Genotype** | **GT Count** | **GT_AdjMean** | **GT_SD** | **Beta** | **P-value** |
| APOE560/rs449647 | 44905307 | 5' flanking | 5 | 0.161 | AA/AT/TT | 433/172/12 | 139.61/134.35/133.8 | 68.2/59.3/67.0 | -0.02 | 0.5229 |
| APOE832/rs405509 | 44905579 | 5' flanking | 1f | 0.4775 | GG/GT/TT | 170/307/141 | 148.79/135.95/129.7 | 71.4/63.4/62.4 | -0.07 | **0.0033** |
| APOE1163/rs440446 | 44905910 | Intron 1 | 4 | 0.3604 | CC/GC/GG | 75/295/249 | 123.14/135.63/145.12 | 56.1/65.4/67.8 | -0.08 | **0.0018** |
| APOE1575/rs769448 | 44906322 | Intron 1 | 4 | 0.021 | CC/CT/TT | 591/24/1 | 138.4/130.89/34.9 | 66.3/48.8/NA | -0.09 | 0.2323 |
| APOE1998/rs769449 | 44906745 | intron 2 | 4 | 0.1165 | AA/AG/GG | 6/131/477 | 179.59/135.92/138 | 80.3/63.7/66.0 | 0.02 | 0.6867 |
| APOE2440/rs769450 | 44907187 | intron 2 | 5 | 0.4015 | AA/GA/GG | 95/305/215 | 148.93/137.7/132.05 | 67.2/64.5/65.2 | 0.06 | **0.0082** |
| APOE2907/rs769451 | 44907654 | intron 2 | 5 | 0.0112 | GT/TT | 14/605 | 124.87/138.24 | 48.8/66.1 | -0.05 | 0.6157 |
| APOE3038/rs111833428 | 44907785 | exon 3 (Ala 23 Ala) | 5 | 0.0016 | AG/GG | 2/612 | 146.64/138.14 | 10.6/66.0 | 0.16 | 0.5766 |
| APOE3106/rs769452 | 44907853 | exon 3 (Leu46Pro) | 5 | 0.0008 | TC/TT | 1/616 | 101.15/137.96 | NA/65.7 | -0.22 | 0.5865 |
| APOE3937/rs429358 | 44908684 | exon 4 (Cys 130 Arg) | 5 | 0.1525 | CC/CT/TT | 14/159/437 | 142.88/138.64/137.37 | 74.6/64.4/65.4 | 0.01 | 0.7069 |
| APOE4075/rs7412 | 44908822 | exon 4 (Arg 176 Cys) | 5 | 0.0806 | CC/TC/TT | 521/92/3 | 137.8/138.76/179.42 | 65.0/70.1/93.6 | 0.01 | 0.7438 |
| APOE4310/rs199768005 | 44909057 | exon 4 (Val 254 Glu) | 5 | 0.004 | TA/TT | 5/613 | 78.12/138.52 | 20.5/65.9 | -0.40 | **0.026** |
| APOE4528/rs374329439 | 44909275 | exon 4 (3' UTR) | 5 | 0.0008 | CC/CT | 618/1 | 137.65/311.89 | 65.5/NA | 0.92 | **0.0232** |
| APOE4737/rs117656888 | 44909484 | 3'flanking | 5 | 0.0081 | CC/GC | 606/10 | 137.88/153.84 | 65.6/79.0 | 0.09 | 0.5084 |
| APOE5361/rs1081106 | 44910109 | 3'flanking | 3a | 0.0852 | CC/TC/TT | 4/98/516 | 186.18/134.11/138.33 | 76.3/65.6/65.7 | -0.006 | 0.8928 |
| **HDL-C^a^** | | | | | | | | | | |
| **Variant name** | **Chr. Position** | **Location** | **RegulomeDB score** | **MAF** | **Genotype** | **GT Count** | **GT_AdjMean** | **GT_SD** | **Beta** | **P-value** |
| APOE560/rs449647 | 44905307 | 5' flanking | 5 | 0.161 | AA/AT/TT | 431/176/12 | 50.95/49.6/50.65 | 14.4/13.4/11.0 | -0.01 | 0.3526 |
| APOE832/rs405509 | 44905579 | 5' flanking | 1f | 0.4775 | GG/GT/TT | 168/310/142 | 50.07/50.56/51.17 | 13.9/14.0/14.2 | 0.01 | 0.4031 |
| APOE1163/rs440446 | 44905910 | Intron 1 | 4 | 0.3604 | CC/GC/GG | 76/297/248 | 51.8/50.73/50.04 | 13.8/14.3/13.7 | 0.01 | 0.2865 |
| APOE1575/rs769448 | 44906322 | Intron 1 | 4 | 0.021 | CC/CT/TT | 593/24/1 | 50.27/55.4/70.05 | 13.8/17.1/NA | 0.08 | **0.0197** |
| APOE1998/rs769449 | 44906745 | intron 2 | 4 | 0.1165 | AA/AG/GG | 6/132/478 | 48.13/50.11/50.79 | 9.6/14.0/14.1 | -0.01 | 0.5800 |
| APOE2440/rs769450 | 44907187 | intron 2 | 5 | 0.4015 | AA/GA/GG | 94/306/217 | 50.42/50.88/50.12 | 15.4/13.5/14.2 | 0.003 | 0.7725 |
| APOE2907/rs769451 | 44907654 | intron 2 | 5 | 0.0112 | GT/TT | 14/607 | 52.84/50.53 | 11.4/14.1 | 0.05 | 0.3390 |
| APOE3038/rs111833428 | 44907785 | exon 3 (Ala 23 Ala) | 5 | 0.0016 | AG/GG | 2/614 | 55.59/50.51 | 4.2/14.1 | 0.10 | 0.4366 |
| APOE3106/rs769452 | 44907853 | exon 3 (Leu46Pro) | 5 | 0.0008 | TC/TT | 1/618 | 61.96/50.57 | NA/14.1 | 0.20 | 0.2787 |
| APOE3937/rs429358 | 44908684 | exon 4 (Cys 130 Arg) | 5 | 0.1525 | CC/CT/TT | 14/159/438 | 47.93/49.89/51.01 | 12.7/14.1/14.1 | -0.02 | 0.2233 |
| APOE4075/rs7412 | 44908822 | exon 4 (Arg 176 Cys) | 5 | 0.0806 | CC/TC/TT | 522/93/3 | 50.76/49.16/54.02 | 14.3/12.6/16.1 | -0.02 | 0.4524 |
| APOE4310/rs199768005 | 44909057 | exon 4 (Val 254 Glu) | 5 | 0.004 | TA/TT | 5/615 | 50.32/50.54 | 10.5/14.0 | 0.01 | 0.8927 |
| APOE4528/rs374329439 | 44909275 | exon 4 (3' UTR) | 5 | 0.0008 | CC/CT | 620/1 | 50.56/64.69 | 14.0/NA | 0.24 | 0.1926 |
| APOE4737/rs117656888 | 44909484 | 3'flanking | 5 | 0.0081 | CC/GC | 608/10 | 50.4/58.73 | 13.9/20.1 | 0.12 | **0.0487** |
| APOE5361/rs1081106 | 44910109 | 3'flanking | 3a | 0.0852 | CC/TC/TT | 4/97/519 | 54.27/52.26/50.19 | 17.2/14.3/13.9 | 0.03 | 0.0915 |

MAF is the minor allele frequency; GT is genotype; GT count is the number of individuals in each genotype group; GT_AdjMean is adjusted mean of lipid trait in each genotype group; GT_SD is standard deviation of lipid traits mean in each genotype group; ^a^ Box-Cox transformed lipid traits.

Table S6. Single-site association analysis in African Blacks (n=788)

| **LDL-C^a^** | | | | | | | | | | |
| --- | --- | --- | --- | --- | --- | --- | --- | --- | --- | --- |
| **Variant name** | **Chr. Position** | **Location** | **RegulomeDB score** | **MAF** | **Genotype** | **GT Count** | **GT_AdjMean** | **GT_SD** | **Beta** | **P-value** |
| APOE73/rs1081101 | 44904820 | 5'flanking | 4 | 0.0611 | CC/CT/TT | 660/82/5 | 109.82/104.38/99.53 | 34.8 /33.9/34.1 | -0.62 | 0.1110 |
| APOE173 | 44904920 | 5'flanking | 3a | 0.0020 | AA/GA | 746/3 | 109.16/99.49 | 34.7/40.5 | -1.14 | 0.5979 |
| APOE308/rs769445 | 44905055 | 5'flanking | 4 | 0.0072 | CC/TC | 739/10 | 109.16/106.69 | 34.6/42.5 | -0.37 | 0.7576 |
| APOE560/rs449647 | 44905307 | 5'flanking | 5 | 0.3663 | AA/AT/TT | 300/334/105 | 105.33/110.93/115.09 | 33.1/36.0/33.4 | 0.58 | **0.0031** |
| APOE618 | 44905365 | 5'flanking | 4 | 0.0006 | GC/GG | 1/763 | 83.39/109.35 | NA/34.5 | -2.85 | 0.4432 |
| APOE624/rs769446 | 44905371 | 5'flanking | 3a | 0.0077 | TC/TT | 9/690 | 85.79/109.43 | 33.9/34.4 | -2.86 | **0.0214** |
| APOE832/rs405509 | 44905579 | 5'flanking | 1f | 0.2561 | GG/GT/TT | 428/267/61 | 105.85/114.09/111.31 | 34.0/36.1/30.7 | 0.60 | **0.0044** |
| APOE1109/rs9282609 | 44905856 | splice site | 4 | 0.0415 | CC/TC/TT | 686/55/4 | 109.01/109/110.06 | 34.7/33.6/31.7 | 0.05 | 0.9183 |
| APOE1163/rs440446 | 44905910 | intron1 | 4 | 0.1004 | CC/CG/GG | 8/126/569 | 106.24/113.53/108.52 | 22.4/36.0/34.7 | 0.44 | 0.1791 |
| APOE1231 | 44905978 | intron1 | 2b | 0.0125 | GA/GG | 19/729 | 102.61/109.3 | 30.1/34.8 | -0.71 | 0.4139 |
| APOE1279/rs877973 | 44906026 | intron1 | 4 | 0.0597 | AA/CA/CC | 3/81/664 | 75.36/111.5/109.08 | 40.0/39.9/34.0 | -0.15 | 0.7208 |
| APOE1539/rs184686013 | 44906286 | intron1 | 4 | 0.0086 | AA/AG/GG | 733/11/1 | 109.19/102.23/77.29 | 34.6/30.1/NA | -1.03 | 0.2862 |
| APOE2072/rs189660912 | 44906819 | intron 2 | 4 | 0.0079 | GA/GG | 12/734 | 103.59/109.27 | 21.4/34.9 | -0.50 | 0.6433 |
| APOE2269/rs61357706 | 44907016 | intron 2 | 5 | 0.0169 | GA/GG | 25/723 | 92.91/110.08 | 30.6/34.6 | -2.05 | **0.0064** |
| APOE2440/rs769450 | 44907187 | intron 2 | 5 | 0.3870 | AA/AG/GG | 107/308/261 | 112.51/109.87/108.31 | 37.6/35.0/33.7 | 0.20 | 0.3409 |
| APOE2544/rs115299243 | 44907291 | intron 2 | 5 | 0.0190 | AA/GA/GG | 599/21/1 | 109.1/90.15/72.71 | 34.6/29.0/NA | -2.34 | **0.0017** |
| APOE3673/rs769453 | 44908420 | intron 3 | 5 | 0.0066 | CC/GC | 738/9 | 109.07/106.76 | 34.4/44.5 | -0.37 | 0.7649 |
| APOE3937/rs429358 | 44908684 | exon 4 (Cys 130 Arg) | 5 | 0.2656 | CC/CT/TT | 58/285/406 | 109.67/113.33/106.49 | 31.8/35.5/34.3 | 0.46 | **0.0317** |
| APOE4036/rs769455 | 44908783 | exon 4 (Arg 163 Cys) | 5 | 0.0200 | CC/TC/TT | 708/27/1 | 109.86/91.6/71.5 | 34.4/28.0/NA | -2.23 | **0.0009** |
| APOE4075/rs7412 | 44908822 | exon 4 (Arg 176 Cys) | 5 | 0.0605 | AA/GA/GG | 2/84/670 | 50.48/95.05/111.16 | 6.8/30.7/34.7 | -2.05 | **5.35E-07** |
| APOE4569 | 44909316 | exon 4 (3'UTR) | 5 | 0.0007 | GG/GT | 746/1 | 109.02/201.25 | 34.6/NA | 8.87 | **0.0173** |
| APOE5223 | 44909970 | 3'flanking | 2b | 0.0051 | CC/CG | 758/8 | 109.45/90.42 | 34.5/28.8 | -2.25 | 0.0874 |
| APOE5231 | 44909978 | 3'flanking | 2b | 0.0270 | GG/GT/TT | 2/36/708 | 93.74/109.69/109.12 | 32.2/36.0/34.7 | -0.10 | 0.8587 |
| **ApoB^a^** | | | | | | | | | | |
| **Variant name** | **Chr. Position** | **Location** | **RegulomeDB score** | **MAF** | **Genotype** | **GT Count** | **GT_AdjMean** | **GT_SD** | **Beta** | **P-value** |
| APOE73/rs1081101 | 44904820 | 5'flanking | 4 | 0.0611 | CC/CT/TT | 658/82/5 | 66.45/67.4/73.43 | 21.5/24.7/7.9 | 0.65 | 0.5488 |
| APOE173 | 44904920 | 5'flanking | 3a | 0.0020 | AA/GA | 744/3 | 66.5/72.76 | 21.8/14.2 | 3.20 | 0.5923 |
| APOE308/rs769445 | 44905055 | 5'flanking | 4 | 0.0072 | CC/TC | 736/11 | 66.47/70.18 | 21.8/21.5 | 1.87 | 0.5514 |
| APOE560/rs449647 | 44905307 | 5'flanking | 5 | 0.3663 | AA/AT/TT | 302/335/105 | 65.41/66.78/69.26 | 22.9/21.9/18.9 | 0.92 | 0.0951 |
| APOE618 | 44905365 | 5'flanking | 4 | 0.0006 | GC/GG | 1/766 | 74.68/66.72 | NA/21.8 | 4.15 | 0.6876 |
| APOE624/rs769446 | 44905371 | 5'flanking | 3a | 0.0077 | TC/TT | 10/693 | 57.76/66.77 | 13.5/21.9 | -4.19 | 0.2037 |
| APOE832/rs405509 | 44905579 | 5'flanking | 1f | 0.2561 | GG/GT/TT | 429/269/61 | 65.72/68.69/66.11 | 21.6/22.1/21.5 | 0.66 | 0.2611 |
| APOE1109/rs9282609 | 44905856 | splice site | 4 | 0.0415 | CC/TC/TT | 684/55/4 | 66.07/71.5/56.8 | 21.4/24.4/28.8 | 1.38 | 0.2768 |
| APOE1163/rs440446 | 44905910 | intron1 | 4 | 0.1004 | CC/CG/GG | 8/126/568 | 73.38/71.01/66.01 | 15.1/22.5/21.6 | 2.30 | **0.0109** |
| APOE1231 | 44905978 | intron1 | 2b | 0.0125 | GA/GG | 19/727 | 62.87/66.6 | 18.1/21.9 | -1.71 | 0.4748 |
| APOE1279/rs877973 | 44906026 | intron1 | 4 | 0.0597 | AA/CA/CC | 4/82/660 | 51.79/67.05/66.53 | 6.6/24.2/21.6 | -0.45 | 0.6847 |
| APOE1539/rs184686013 | 44906286 | intron1 | 4 | 0.0086 | AA/AG/GG | 732/11/1 | 66.43/70.12/48.6 | 21.8/20.5/NA | 0.23 | 0.9330 |
| APOE2072/rs189660912 | 44906819 | intron 2 | 4 | 0.0079 | GA/GG | 12/732 | 67.45/66.55 | 10.5/22.0 | 0.70 | 0.8149 |
| APOE2269/rs61357706 | 44907016 | intron 2 | 5 | 0.0169 | GA/GG | 25/726 | 61.69/66.98 | 19.9/21.8 | -2.56 | 0.2201 |
| APOE2440/rs769450 | 44907187 | intron 2 | 5 | 0.3870 | AA/AG/GG | 109/302/263 | 65.5/67.83/67.87 | 22.4/21.8/20.9 | -0.50 | 0.3725 |
| APOE2544/rs115299243 | 44907291 | intron 2 | 5 | 0.0190 | AA/GA/GG | 598/22/1 | 65.52/57.67/48.57 | 21.8/22.6/NA | -4.01 | 0.0524 |
| APOE3673/rs769453 | 44908420 | intron 3 | 5 | 0.0066 | CC/GC | 735/10 | 66.4/70.47 | 21.7/22.5 | 2.02 | 0.5386 |
| APOE3937/rs429358 | 44908684 | exon 4 (Cys 130 Arg) | 5 | 0.2656 | CC/CT/TT | 56/288/408 | 65.25/67.46/66.46 | 20.4/22.6/21.6 | 0.05 | 0.9371 |
| APOE4036/rs769455 | 44908783 | exon 4 (Arg 163 Cys) | 5 | 0.0200 | CC/TC/TT | 706/27/1 | 66.89/60.39/48.19 | 21.7/22.7/NA | -3.45 | 0.0664 |
| APOE4075/rs7412 | 44908822 | exon 4 (Arg 176 Cys) | 5 | 0.0605 | AA/GA/GG | 3/84/672 | 45.96/63.02/67.16 | 27.7/18.2/22.2 | -2.35 | **0.0356** |
| APOE4569 | 44909316 | exon 4 (3'UTR) | 5 | 0.0007 | GG/GT | 744/1 | 66.46/96.65 | 21.8/NA | 14.25 | 0.1684 |
| APOE5223 | 44909970 | 3'flanking | 2b | 0.0051 | CC/CG | 762/7 | 66.75/61.28 | 21.9/17.7 | -2.54 | 0.5166 |
| APOE5231 | 44909978 | 3'flanking | 2b | 0.0270 | GG/GT/TT | 2/37/705 | 50.22/66.27/66.55 | 17.5/21.6/21.9 | -0.82 | 0.6036 |
| **TG^a^** | | | | | | | | | | |
| **Variant name** | **Chr. Position** | **Location** | **RegulomeDB score** | **MAF** | **Genotype** | **GT Count** | **GT_AdjMean** | **GT_SD** | **Beta** | **P-value** |
| APOE73/rs1081101 | 44904820 | 5'flanking | 4 | 0.0611 | CC/CT/TT | 659/82/5 | 69.19/76.65/92.6 | 29.8/34.0/55.5 | 0.038 | **0.0093** |
| APOE173 | 44904920 | 5'flanking | 3a | 0.0020 | AA/GA | 745/2 | 70.13/73.71 | 30.6/30.4 | 0.037 | 0.7028 |
| APOE308/rs769445 | 44905055 | 5'flanking | 4 | 0.0072 | CC/TC | 737/10 | 70.03/77.84 | 30.7/23.3 | 0.056 | 0.2065 |
| APOE560/rs449647 | 44905307 | 5'flanking | 5 | 0.3663 | AA/AT/TT | 301/335/102 | 70.73/69.69/70.08 | 31.6/28.9/31.7 | -0.001 | 0.8482 |
| APOE618 | 44905365 | 5'flanking | 4 | 0.0006 | GC/GG | 1/760 | 68.05/70.26 | NA/30.5 | 0.025 | 0.8560 |
| APOE624/rs769446 | 44905371 | 5'flanking | 3a | 0.0077 | TC/TT | 11/687 | 69.92/70.44 | 27.6/30.6 | 0.005 | 0.9096 |
| APOE832/rs405509 | 44905579 | 5'flanking | 1f | 0.2561 | GG/GT/TT | 427/268/59 | 69.98/71.39/68.15 | 31.2/30.0/30.0 | 0.003 | 0.7457 |
| APOE1109/rs9282609 | 44905856 | splice site | 4 | 0.0415 | CC/TC/TT | 685/55/4 | 69.83/73.46/88.67 | 30.5/28.6/66.8 | 0.023 | 0.1814 |
| APOE1163/rs440446 | 44905910 | intron1 | 4 | 0.1004 | CC/CG/GG | 8/126/569 | 92.59/72.71/69.62 | 59.4/28.8/30.5 | 0.023 | 0.0537 |
| APOE1231 | 44905978 | intron1 | 2b | 0.0125 | GA/GG | 19/727 | 67.34/70.14 | 38.4/30.4 | -0.030 | 0.3441 |
| APOE1279/rs877973 | 44906026 | intron1 | 4 | 0.0597 | AA/CA/CC | 3/82/661 | 60.01/64.63/70.92 | 23.2/26.1/31.1 | -0.029 | 0.0513 |
| APOE1539/rs184686013 | 44906286 | intron1 | 4 | 0.0086 | AA/AG/GG | 732/11/1 | 70.28/66.57/80.4 | 30.7/27.1/NA | 0.002 | 0.9546 |
| APOE2072/rs189660912 | 44906819 | intron 2 | 4 | 0.0079 | GA/GG | 12/733 | 68.98/70.12 | 25.0/30.7 | 0.009 | 0.8229 |
| APOE2269/rs61357706 | 44907016 | intron 2 | 5 | 0.0169 | GA/GG | 25/722 | 79.21/69.84 | 37.5/30.0 | 0.040 | 0.1489 |
| APOE2440/rs769450 | 44907187 | intron 2 | 5 | 0.3870 | AA/AG/GG | 107/309/261 | 69.13/70.46/70.84 | 29.1/30.7/31.3 | -0.003 | 0.7396 |
| APOE2544/rs115299243 | 44907291 | intron 2 | 5 | 0.0190 | AA/GA | 598/21 | 70.17/83.63 | 30.3/37.7 | 0.061 | **0.0470** |
| APOE3673/rs769453 | 44908420 | intron 3 | 5 | 0.0066 | CC/GC | 737/9 | 70.23/74.25 | 30.7/23.4 | 0.037 | 0.4242 |
| APOE3937/rs429358 | 44908684 | exon 4 (Cys 130 Arg) | 5 | 0.2656 | CC/CT/TT | 56/287/405 | 67.14/69.52/70.98 | 27.5/30.9/30.7 | -0.008 | 0.3075 |
| APOE4036/rs769455 | 44908783 | exon 4 (Arg 163 Cys) | 5 | 0.0200 | CC/TC | 709/27 | 69.45/83.67 | 29.8/42.8 | 0.056 | **0.0372** |
| APOE4075/rs7412 | 44908822 | exon 4 (Arg 176 Cys) | 5 | 0.0605 | AA/GA/GG | 3/85/666 | 49.67/68.01/70.51 | 16.9/29.1/30.8 | -0.018 | 0.2376 |
| APOE4569 | 44909316 | exon 4 (3'UTR) | 5 | 0.0007 | GG/GT | 744/1 | 70.08/77.54 | 30.6/NA | 0.073 | 0.5976 |
| APOE5223 | 44909970 | 3'flanking | 2b | 0.0051 | CC/CG | 756/8 | 70.38/70.64 | 30.7/29.6 | 0.006 | 0.9078 |
| APOE5231 | 44909978 | 3'flanking | 2b | 0.0270 | GG/GT/TT | 2/35/707 | 68.24/66.1/70.39 | 17.7/32.2/30.6 | -0.023 | 0.2836 |
| **HDL-C^a^** | | | | | | | | | | |
| **Variant name** | **Chr. Position** | **Location** | **RegulomeDB score** | **MAF** | **Genotype** | **GT Count** | **GT_AdjMean** | **GT_SD** | **Beta** | **P-value** |
| APOE73/rs1081101 | 44904820 | 5'flanking | 4 | 0.0611 | CC/CT/TT | 652/82/5 | 47.72/49.03/48.5 | 12.6/13.7/5.6 | 0.30 | 0.4273 |
| APOE173 | 44904920 | 5'flanking | 3a | 0.0020 | AA/GA | 738/3 | 47.84/40.69 | 12.7/11.2 | -2.10 | 0.3206 |
| APOE308/rs769445 | 44905055 | 5'flanking | 4 | 0.0072 | CC/TC | 730/11 | 47.86/44.21 | 12.7/11.0 | -1.06 | 0.3428 |
| APOE560/rs449647 | 44905307 | 5'flanking | 5 | 0.3663 | AA/AT/TT | 296/333/103 | 48.06/47.85/46.63 | 12.5/13.0/12.7 | -0.18 | 0.3564 |
| APOE618 | 44905365 | 5'flanking | 4 | 0.0006 | GC/GG | 1/754 | 13.45/47.78 | NA/12.6 | -12.25 | **0.0008** |
| APOE624/rs769446 | 44905371 | 5'flanking | 3a | 0.0077 | TC/TT | 11/681 | 49.89/47.71 | 15.1/12.9 | 0.65 | 0.5687 |
| APOE832/rs405509 | 44905579 | 5'flanking | 1f | 0.2561 | GG/GT/TT | 421/268/59 | 47.91/48.13/45.19 | 12.9/12.7/11.3 | -0.19 | 0.3612 |
| APOE1109/rs9282609 | 44905856 | splice site | 4 | 0.0415 | CC/TC/TT | 679/54/4 | 47.63/50.15/49.36 | 12.6/13.7/6.6 | 0.61 | 0.1788 |
| APOE1163/rs440446 | 44905910 | intron1 | 4 | 0.1004 | CC/CG/GG | 8/126/562 | 45.27/46.49/48.41 | 14.2/12.5/12.8 | -0.54 | 0.0946 |
| APOE1231 | 44905978 | intron1 | 2b | 0.0125 | GA/GG | 18/722 | 46.84/47.87 | 11.5/12.7 | -0.27 | 0.7550 |
| APOE1279/rs877973 | 44906026 | intron1 | 4 | 0.0597 | AA/CA/CC | 4/79/657 | 49.78/49.36/47.61 | 11.2/12.3/12.7 | 0.51 | 0.1981 |
| APOE1539/rs184686013 | 44906286 | intron1 | 4 | 0.0086 | AA/AG/GG | 725/11/1 | 47.68/54.07/46.37 | 12.7/13.5/NA | 1.35 | 0.1568 |
| APOE2072/rs189660912 | 44906819 | intron 2 | 4 | 0.0079 | GA/GG | 12/726 | 46.66/47.85 | 15.0/12.7 | -0.40 | 0.7066 |
| APOE2269/rs61357706 | 44907016 | intron 2 | 5 | 0.0169 | GA/GG | 26/714 | 50.39/47.78 | 12.6/12.7 | 0.77 | 0.2912 |
| APOE2440/rs769450 | 44907187 | intron 2 | 5 | 0.3870 | AA/AG/GG | 106/303/260 | 46.94/48.89/47.64 | 13.4/13.3/12.3 | -0.02 | 0.9394 |
| APOE2544/rs115299243 | 44907291 | intron 2 | 5 | 0.0190 | AA/GA/GG | 592/22/1 | 47.71/49.06/15.6 | 13.2/13.8/NA | -0.54 | 0.4827 |
| APOE3673/rs769453 | 44908420 | intron 3 | 5 | 0.0066 | CC/GC | 729/10 | 47.85/44.06 | 12.7/11.5 | -1.10 | 0.3457 |
| APOE3937/rs429358 | 44908684 | exon 4 (Cys 130 Arg) | 5 | 0.2656 | CC/CT/TT | 57/284/400 | 46.45/47.78/48 | 12.5/12.0/13.3 | -0.14 | 0.5074 |
| APOE4036/rs769455 | 44908783 | exon 4 (Arg 163 Cys) | 5 | 0.0200 | CC/TC/TT | 700/28/1 | 47.95/48.87/15.7 | 12.7/12.5/NA | -0.46 | 0.4827 |
| APOE4075/rs7412 | 44908822 | exon 4 (Arg 176 Cys) | 5 | 0.0605 | AA/GA/GG | 2/84/662 | 50.84/49.96/47.54 | 12.7/11.5/12.8 | 0.75 | 0.0661 |
| APOE4569 | 44909316 | exon 4 (3'UTR) | 5 | 0.0007 | GG/GT | 738/1 | 47.84/66.63 | 12.7/NA | 5.29 | 0.1497 |
| APOE5223 | 44909970 | 3'flanking | 2b | 0.0051 | CC/CG | 750/8 | 47.74/48.97 | 12.7/12.1 | 0.39 | 0.7630 |
| APOE5231 | 44909978 | 3'flanking | 2b | 0.0270 | GG/GT/TT | 1/35/702 | 51.97/46.78/47.88 | NA/11.1/12.8 | -0.18 | 0.7645 |

MAF is the minor allele frequency; GT is genotype; GT count is the number of individuals in each genotype group; GT_AdjMean is the adjusted mean of the lipid trait in each genotype group; GT_SD is the standard deviation of the lipid traits mean in each genotype group; ^a^ Box-Cox transformed lipid traits.

Table S7. 4-SNPs window haplotype-based association results for LDL-C, TG and HDL-C in NHWs (n=623)

|  |  |  |  |  | **LDL-C** | | | **TG^a^** | | | **HDL-C^a^** | | |
| --- | --- | --- | --- | --- | --- | --- | --- | --- | --- | --- | --- | --- | --- |
| **wind** | **w.snp** | **Location** | **Genotype** | **MAF** | **Beta** | **p_value** | **hap_P** | **Beta** | **p_value** | **hap_P** | **Beta** | **p_value** | **hap_P** |
| 1 | APOE560/rs449647 | 5' flanking | AA/AT/TT | 0.1610 | -7.12 | 0.0247 | **0.0089** | -0.02 | 0.5229 | **0.0043** | -0.01 | 0.3526 | 0.0873 |
| 1 | APOE832/rs405509 | 5' flanking | GG/GT/TT | 0.4775 | 0.42 | 0.8542 | NA | -0.07 | 0.0033 | NA | 0.01 | 0.4031 | NA |
| 1 | APOE1163/rs440446 | intron 1 | CC/GC/GG | 0.3604 | -2.59 | 0.2810 | NA | -0.08 | 0.0018 | NA | 0.01 | 0.2865 | NA |
| 1 | APOE1575/rs769448 | intron 1 | CC/CT/TT | 0.0210 | 0.75 | 0.9232 | NA | -0.09 | 0.2323 | NA | 0.08 | 0.0197 | NA |
| 2 | APOE832/rs405509 | 5' flanking | GG/GT/TT | 0.4775 | 0.42 | 0.8542 | 0.3923 | -0.07 | 0.0033 | **0.0196** | 0.01 | 0.4031 | 0.2088 |
| 2 | APOE1163/rs440446 | intron 1 | CC/GC/GG | 0.3604 | -2.59 | 0.2810 | NA | -0.08 | 0.0018 | NA | 0.01 | 0.2865 | NA |
| 2 | APOE1575/rs769448 | intron 1 | CC/CT/TT | 0.0210 | 0.75 | 0.9232 | NA | -0.09 | 0.2323 | NA | 0.08 | 0.0197 | NA |
| 2 | APOE1998/rs769449 | intron 2 | AA/AG/GG | 0.1165 | 6.95 | 0.0551 | NA | 0.02 | 0.6867 | NA | -0.01 | 0.5800 | NA |
| 3 | APOE1163/rs440446 | intron 1 | CC/GC/GG | 0.3604 | -2.59 | 0.2810 | **0.0035** | -0.08 | 0.0018 | **0.0194** | 0.01 | 0.2865 | 0.1047 |
| 3 | APOE1575/rs769448 | intron 1 | CC/CT/TT | 0.0210 | 0.75 | 0.9232 | NA | -0.09 | 0.2323 | NA | 0.08 | 0.0197 | NA |
| 3 | APOE1998/rs769449 | intron 2 | AA/AG/GG | 0.1165 | 6.95 | 0.0551 | NA | 0.02 | 0.6867 | NA | -0.01 | 0.5800 | NA |
| 3 | APOE2440/rs769450 | intron 2 | AA/GA/GG | 0.4015 | 4.89 | 0.0378 | NA | 0.06 | 0.0082 | NA | 0.003 | 0.7725 | NA |
| 4 | APOE1575/rs769448 | intron 1 | CC/CT/TT | 0.0210 | 0.75 | 0.9232 | **0.0183** | -0.09 | 0.2323 | 0.0503 | 0.08 | 0.0197 | 0.1220 |
| 4 | APOE1998/rs769449 | intron 2 | AA/AG/GG | 0.1165 | 6.95 | 0.0551 | NA | 0.02 | 0.6867 | NA | -0.01 | 0.5800 | NA |
| 4 | APOE2440/rs769450 | intron 2 | AA/GA/GG | 0.4015 | 4.89 | 0.0378 | NA | 0.06 | 0.0082 | NA | 0.003 | 0.7725 | NA |
| 4 | APOE2907rs769451 | intron 2 | GT/TT | 0.0112 | 0.45 | 0.9668 | NA | -0.05 | 0.6157 | NA | 0.05 | 0.3390 | NA |
| 5 | APOE1998/rs769449 | intron 2 | AA/AG/GG | 0.1165 | 6.95 | 0.0551 | **0.0101** | 0.02 | 0.6867 | **0.0344** | -0.01 | 0.5800 | 0.6275 |
| 5 | APOE2440/rs769450 | intron 2 | AA/GA/GG | 0.4015 | 4.89 | 0.0378 | NA | 0.06 | 0.0082 | NA | 0.003 | 0.7725 | NA |
| 5 | APOE2907/rs769451 | intron 2 | GT/TT | 0.0112 | 0.45 | 0.9668 | NA | -0.05 | 0.6157 | NA | 0.05 | 0.3390 | NA |
| 5 | APOE3038/rs111833428 | exon 3 (Ala 23 Ala) | AG/GG | 0.0016 | 27.34 | 0.3354 | NA | 0.16 | 0.5766 | NA | 0.10 | 0.4366 | NA |
| 6 | APOE2440/rs769450 | intron 2 | AA/GA/GG | 0.4015 | 4.89 | 0.0378 | 0.1519 | 0.06 | 0.0082 | 0.0708 | 0.003 | 0.7725 | 0.4432 |
| 6 | APOE2907/rs769451 | intron 2 | GT/TT | 0.0112 | 0.45 | 0.9668 | NA | -0.05 | 0.6157 | NA | 0.05 | 0.3390 | NA |
| 6 | APOE3038/rs111833428 | exon 3 (Ala 23 Ala) | AG/GG | 0.0016 | 27.34 | 0.3354 | NA | 0.16 | 0.5766 | NA | 0.10 | 0.4366 | NA |
| 6 | APOE3106/rs769452 | exon 3 (Leu46Pro) | TC/TT | 0.0008 | 18.03 | 0.6522 | NA | -0.22 | 0.5865 | NA | 0.20 | 0.2787 | NA |
| 7 | APOE2907/rs769451 | intron 2 | GT/TT | 0.0112 | 0.45 | 0.9668 | **0.0339** | -0.05 | 0.6157 | 0.8488 | 0.05 | 0.3390 | 0.1637 |
| 7 | APOE3038/rs111833428 | exon 3 (Ala 23 Ala) | AG/GG | 0.0016 | 27.34 | 0.3354 | NA | 0.16 | 0.5766 | NA | 0.10 | 0.4366 | NA |
| 7 | APOE3106/rs769452 | exon 3 (Leu46Pro) | TC/TT | 0.0008 | 18.03 | 0.6522 | NA | -0.22 | 0.5865 | NA | 0.20 | 0.2787 | NA |
| 7 | APOE3937/rs429358 | exon 4 (Cys 130 Arg) | CC/CT/TT | 0.1525 | 8.10 | 0.0103 | NA | 0.01 | 0.7069 | NA | -0.02 | 0.2233 | NA |
| 8 | APOE3038/rs111833428 | exon 3 (Ala 23 Ala) | AG/GG | 0.0016 | 27.34 | 0.3354 | **2.03E-07** | 0.16 | 0.5766 | 0.9439 | 0.10 | 0.4366 | 0.2664 |
| 8 | APOE3106/rs769452 | exon 3 (Leu46Pro) | TC/TT | 0.0008 | 18.03 | 0.6522 | NA | -0.22 | 0.5865 | NA | 0.20 | 0.2787 | NA |
| 8 | APOE3937/rs429358 | exon 4 (Cys 130 Arg) | CC/CT/TT | 0.1525 | 8.10 | 0.0103 | NA | 0.01 | 0.7069 | NA | -0.02 | 0.2233 | NA |
| 8 | APOE4075/rs7412 | exon 4 (Arg 176 Cys) | CC/TC/TT | 0.0806 | -21.84 | 1.84E-07 | NA | 0.01 | 0.7438 | NA | -0.02 | 0.4524 | NA |
| 9 | APOE3106/rs769452 | exon 3 (Leu46Pro) | TC/TT | 0.0008 | 18.03 | 0.6522 | **2.09E-07** | -0.22 | 0.5865 | 0.1491 | 0.20 | 0.2787 | 0.4221 |
| 9 | APOE3937/rs429358 | exon 4 (Cys 130 Arg) | CC/CT/TT | 0.1525 | 8.10 | 0.0103 | NA | 0.01 | 0.7069 | NA | -0.02 | 0.2233 | NA |
| 9 | APOE4075/rs7412 | exon 4 (Arg 176 Cys) | CC/TC/TT | 0.0806 | -21.84 | 1.84E-07 | NA | 0.01 | 0.7438 | NA | -0.02 | 0.4524 | NA |
| 9 | APOE4310/rs199768005 | exon 4 (Val 254 Glu) | TA/TT | 0.0040 | -32.56 | 0.0705 | NA | -0.40 | 0.0260 | NA | 0.01 | 0.8927 | NA |
| 10 | APOE3937/rs429358 | exon 4 (Cys 130 Arg) | CC/CT/TT | 0.1525 | 8.10 | 0.0103 | **1.12E-07** | 0.01 | 0.7069 | 0.6934 | -0.02 | 0.2233 | 0.4466 |
| 10 | APOE4075/rs7412 | exon 4 (Arg 176 Cys) | CC/TC/TT | 0.0806 | -21.84 | 1.84E-07 | NA | 0.01 | 0.7438 | NA | -0.02 | 0.4524 | NA |
| 10 | APOE4310/rs199768005 | exon 4 (Val 254 Glu) | TA/TT | 0.0040 | -32.56 | 0.0705 | NA | -0.40 | 0.0260 | NA | 0.01 | 0.8927 | NA |
| 10 | APOE4528/rs374329439 | exon 4 (3' UTR) | CC/CT | 0.0008 | -12.30 | 0.7585 | NA | 0.92 | 0.0232 | NA | 0.24 | 0.1926 | NA |
| 11 | APOE4075/rs7412 | exon 4 (Arg 176 Cys) | CC/TC/TT | 0.0806 | -21.84 | 1.84E-07 | **1.02E-06** | 0.01 | 0.7438 | 0.9423 | -0.02 | 0.4524 | 0.1179 |
| 11 | APOE4310/rs199768005 | exon 4 (Val 254 Glu) | TA/TT | 0.0040 | -32.56 | 0.0705 | NA | -0.40 | 0.0260 | NA | 0.01 | 0.8927 | NA |
| 11 | APOE4528/rs374329439 | exon 4 (3' UTR) | CC/CT | 0.0008 | -12.30 | 0.7585 | NA | 0.92 | 0.0232 | NA | 0.24 | 0.1926 | NA |
| 11 | APOE4737/rs117656888 | 3'flanking | CC/GC | 0.0081 | 13.58 | 0.2867 | NA | 0.09 | 0.5084 | NA | 0.12 | 0.0487 | NA |
| 12 | APOE4310/rs199768005 | exon 4 (Val 254 Glu) | TA/TT | 0.0040 | -32.56 | 0.0705 | 0.5719 | -0.40 | 0.0260 | 0.9232 | 0.01 | 0.8927 | **0.0301** |
| 12 | APOE4528/rs374329439 | exon 4 (3' UTR) | CC/CT | 0.0008 | -12.30 | 0.7585 | NA | 0.92 | 0.0232 | NA | 0.24 | 0.1926 | NA |
| 12 | APOE4737/rs117656888 | 3'flanking | CC/GC | 0.0081 | 13.58 | 0.2867 | NA | 0.09 | 0.5084 | NA | 0.12 | 0.0487 | NA |
| 12 | APOE5361/rs1081106 | 3'flanking | CC/TC/TT | 0.0852 | 4.00 | 0.3272 | NA | -0.006 | 0.8928 | NA | 0.03 | 0.0915 | NA |

wind: 4-SNPs haplotype window; w.snps: SNPs that were included in each window; p-value: single-site p-value; hap-P: haplotype global p-value; ^a^ Box-Cox transformed data.

Table S8. 4-SNPs window haplotype-based association results for ApoB in NHWs (n=623)

| **ApoB^a^** | | | | | | | |
| --- | --- | --- | --- | --- | --- | --- | --- |
| **wind** | **w.snp** | **Location** | **Genotype** | **MAF** | **Beta** | **p_value** | **hap_P** |
| 1 | APOE560/rs449647 | 5' flanking | AA/AT/TT | 0.1610 | -0.95 | 0.1290 | **1.57E-05** |
| 1 | APOE832/rs405509 | 5' flanking | GG/GT/TT | 0.4775 | 1.46 | 0.0009 | NA |
| 1 | APOE1163/rs440446 | Intron 1 | CC/GC/GG | 0.3604 | 0.56 | 0.2319 | NA |
| 1 | APOE1575/rs769448 | Intron 1 | CC/CT/TT | 0.0210 | 0.94 | 0.5204 | NA |
| 2 | APOE832/rs405509 | 5' flanking | GG/GT/TT | 0.4775 | 1.46 | 0.0009 | **0.0004** |
| 2 | APOE1163/rs440446 | Intron 1 | CC/GC/GG | 0.3604 | 0.56 | 0.2319 | NA |
| 2 | APOE1575/rs769448 | Intron 1 | CC/CT/TT | 0.0210 | 0.94 | 0.5204 | NA |
| 2 | APOE1998/rs769449 | intron 2 | AA/AG/GG | 0.1165 | 2.03 | 0.0030 | NA |
| 3 | APOE1163/rs440446 | Intron 1 | CC/GC/GG | 0.3604 | 0.56 | 0.2319 | **8.05E-07** |
| 3 | APOE1575/rs769448 | Intron 1 | CC/CT/TT | 0.0210 | 0.94 | 0.5204 | NA |
| 3 | APOE1998/rs769449 | intron 1 | AA/AG/GG | 0.1165 | 2.03 | 0.0030 | NA |
| 3 | APOE2440/rs769450 | intron 2 | AA/GA/GG | 0.4015 | 0.15 | 0.7422 | NA |
| 4 | APOE1575/rs769448 | Intron 1 | CC/CT/TT | 0.0210 | 0.94 | 0.5204 | **0.0265** |
| 4 | APOE1998/rs769449 | intron 1 | AA/AG/GG | 0.1165 | 2.03 | 0.0030 | NA |
| 4 | APOE2440/rs769450 | intron 2 | AA/GA/GG | 0.4015 | 0.15 | 0.7422 | NA |
| 4 | APOE2907/rs769451 | intron 2 | GT/TT | 0.0112 | -0.22 | 0.9196 | NA |
| 5 | APOE1998/rs769449 | intron 1 | AA/AG/GG | 0.1165 | 2.03 | 0.0030 | **0.0176** |
| 5 | APOE2440/rs769450 | intron 2 | AA/GA/GG | 0.4015 | 0.15 | 0.7422 | NA |
| 5 | APOE2907/rs769451 | intron 2 | GT/TT | 0.0112 | -0.22 | 0.9196 | NA |
| 5 | APOE3038/rs111833428 | exon 3 (Ala 23 Ala) | AG/GG | 0.0016 | 13.81 | 0.0342 | NA |
| 6 | APOE2440/rs769450 | intron 2 | AA/GA/GG | 0.4015 | 0.15 | 0.7422 | 0.0965 |
| 6 | APOE2907/rs769451 | intron 2 | GT/TT | 0.0112 | -0.22 | 0.9196 | NA |
| 6 | APOE3038/rs111833428 | exon 3 (Ala 23 Ala) | AG/GG | 0.0016 | 13.81 | 0.0342 | NA |
| 6 | APOE3106/rs769452 | exon 3 (Leu46Pro) | TC/TT | 0.0008 | 9.15 | 0.1602 | NA |
| 7 | APOE2907/rs769451 | intron 2 | GT/TT | 0.0112 | -0.22 | 0.9196 | **0.0027** |
| 7 | APOE3038/rs111833428 | exon 3 (Ala 23 Ala) | AG/GG | 0.0016 | 13.81 | 0.0342 | NA |
| 7 | APOE3106/rs769452 | exon 3 (Leu46Pro) | TC/TT | 0.0008 | 9.15 | 0.1602 | NA |
| 7 | APOE3937/rs429358 | exon 4 (Cys 130 Arg) | CC/CT/TT | 0.1525 | 2.14 | 0.0005 | NA |
| 8 | APOE3038/rs111833428 | exon 3 (Ala 23 Ala) | AG/GG | 0.0016 | 13.81 | 0.0342 | **4.37E-14** |
| 8 | APOE3106/rs769452 | exon 3 (Leu46Pro) | TC/TT | 0.0008 | 9.15 | 0.1602 | NA |
| 8 | APOE3937/rs429358 | exon 4 (Cys 130 Arg) | CC/CT/TT | 0.1525 | 2.14 | 0.0005 | NA |
| 8 | APOE4075/rs7412 | exon 4 (Arg 176 Cys) | CC/TC/TT | 0.0806 | -5.60 | 9.65E-13 | NA |
| 9 | APOE3106/rs769452 | exon 3 (Leu46Pro) | TC/TT | 0.0008 | 9.15 | 0.1602 | **8.32E-13** |
| 9 | APOE3937/rs429358 | exon 4 (Cys 130 Arg) | CC/CT/TT | 0.1525 | 2.14 | 0.0005 | NA |
| 9 | APOE4075/rs7412 | exon 4 (Arg 176 Cys) | CC/TC/TT | 0.0806 | -5.60 | 9.65E-13 | NA |
| 9 | APOE4310/rs199768005 | exon 4 (Val 254 Glu) | TA/TT | 0.0040 | 1.05 | 0.8220 | NA |
| 10 | APOE3937/rs429358 | exon 4 (Cys 130 Arg) | CC/CT/TT | 0.1525 | 2.14 | 0.0005 | **5.47E-12** |
| 10 | APOE4075/rs7412 | exon 4 (Arg 176 Cys) | CC/TC/TT | 0.0806 | -5.60 | 9.65E-13 | NA |
| 10 | APOE4310/rs199768005 | exon 4 (Val 254 Glu) | TA/TT | 0.0040 | 1.05 | 0.8220 | NA |
| 10 | APOE5361/rs1081106 | 3'flanking | CC/TC/TT | 0.0852 | 0.12 | 0.8813 | NA |

wind: 4-SNPs haplotype window; w.snps: SNPs that were included in each window; p-value: single-site p-value; hap-P: haplotype global p-value. ^a^ Box-Cox transformed data.

Table S9. Haplotype-based association summary of significant windows with LDL-C in NHWs (n=623)

| **LDL-C** | | | | | | | | | | |
| --- | --- | --- | --- | --- | --- | --- | --- | --- | --- | --- |
|  | **Window** | **loc.1** | **loc.2** | **loc.3** | **loc.4** | **hap.freq** | **coef** | **se** | **t.stat** | **pval** |
| Geno.3 | 1 | A | T | C | C | 0.2591 | -6.76 | 3.01 | -2.25 | 0.02486 |
| Geno.4 | 1 | A | T | C | T | 0.0211 | -1.35 | 7.63 | -0.18 | 0.85966 |
| Geno.5 | 1 | A | T | G | C | 0.1182 | 3.48 | 3.70 | 0.94 | 0.34709 |
| Geno.7 | 1 | T | G | G | C | 0.0814 | -18.23 | 4.88 | -3.74 | 0.00020 |
| Geno.8 | 1 | T | T | C | C | 0.0783 | 0.97 | 4.57 | 0.21 | 0.83201 |
| Geno.rare | 1 | * | * | * | * | 0.0027 | -18.70 | 0.03 | -616.00 | <10E-06 |
| haplo.base | 1 | A | G | G | C | 0.4391 | NA | NA | NA | NA |
| Geno.2 | 3 | C | C | G | G | 0.3374 | -4.93 | 2.65 | -1.86 | 0.06304 |
| Geno.31 | 3 | C | T | G | G | 0.0211 | -1.67 | 7.67 | -0.22 | 0.82803 |
| Geno.52 | 3 | G | C | A | G | 0.1150 | 2.98 | 3.79 | 0.79 | 0.43109 |
| Geno.72 | 3 | G | C | G | G | 0.1221 | -13.46 | 3.69 | -3.65 | 0.00029 |
| Geno.rare2 | 3 | * | * | * | * | 0.0019 | -10.87 | 0.09 | -124.49 | <10E-06 |
| haplo.base2 | 3 | G | C | G | A | 0.4026 | NA | NA | NA | NA |
| Geno.32 | 4 | C | A | G | T | 0.1150 | 10.18 | 3.78 | 2.69 | 0.00724 |
| Geno.53 | 4 | C | G | A | T | 0.4019 | 7.14 | 2.48 | 2.87 | 0.00419 |
| Geno.9 | 4 | T | G | G | T | 0.0210 | 5.04 | 7.73 | 0.65 | 0.51461 |
| Geno.rare3 | 4 | * | * | * | * | 0.0112 | 3.41 | 10.84 | 0.31 | 0.75341 |
| haplo.base3 | 4 | C | G | G | T | 0.4508 | NA | NA | NA | NA |
| Geno.33 | 5 | A | G | T | G | 0.1147 | 9.92 | 3.76 | 2.64 | 0.00850 |
| Geno.61 | 5 | G | A | T | G | 0.4002 | 6.75 | 2.44 | 2.76 | 0.00586 |
| Geno.rare4 | 5 | * | * | * | * | 0.0129 | 6.44 | 0.24 | 26.92 | <10E-06 |
| haplo.base4 | 5 | G | G | T | G | 0.4722 | NA | NA | NA | NA |
| Geno.62 | 7 | T | G | T | C | 0.1489 | 8.17 | 3.18 | 2.57 | 0.0105 |
| Geno.rare6 | 7 | * | * | * | * | 0.0137 | 6.30 | 0.11 | 55.65 | <10E-06 |
| haplo.base6 | 7 | T | G | T | T | 0.8375 | NA | NA | NA | NA |
| Geno.35 | 8 | G | T | C | C | 0.1490 | 5.03 | 3.20 | 1.57 | 0.11629 |
| Geno.63 | 8 | G | T | T | T | 0.0781 | -22.79 | 4.35 | -5.24 | 2.26E-07 |
| Geno.rare7 | 8 | * | * | * | * | 0.0048 | 40.70 | 0.05 | 853.87 | <10E-06 |
| haplo.base7 | 8 | G | T | T | C | 0.7680 | NA | NA | NA | NA |
| Geno.36 | 9 | T | C | C | T | 0.1514 | 5.97 | 3.12 | 1.92 | 0.05594 |
| Geno.73 | 9 | T | T | T | T | 0.0805 | -20.88 | 4.13 | -5.06 | 5.62E-07 |
| Geno.rare8 | 9 | * | * | * | * | 0.0048 | -26.44 | 0.06 | -427.62 | <10E-06 |
| haplo.base8 | 9 | T | T | C | T | 0.7632 | NA | NA | NA | NA |
| Geno.21 | 10 | C | C | T | C | 0.1522 | 6.01 | 3.11 | 1.93 | 0.05361 |
| Geno.74 | 10 | T | T | T | C | 0.0805 | -20.91 | 4.12 | -5.07 | 5.27E-07 |
| Geno.rare9 | 10 | * | * | * | * | 0.0048 | -31.49 | 0.06 | -507.97 | <10E-06 |
| haplo.base9 | 10 | T | C | T | C | 0.7624 | NA | NA | NA | NA |
| Geno.54 | 11 | T | T | C | C | 0.0805 | -21.80 | 4.12 | -5.29 | 1.68E-07 |
| Geno.rare10 | 11 | * | * | * | * | 0.0129 | -6.08 | 10.20 | -0.60 | 0.55144 |
| haplo.base10 | 11 | C | T | C | C | 0.9066 | NA | NA | NA | NA |

hap.freq: haplotype frequency; coef: coefficient; se: standard error; t.stat: test statistic; p-val: haplotype p-value

Table S10. Haplotype-based association summary of significant windows with ApoB in NHWs (n=623)

| **ApoB^a^** | | | | | | | | | | |
| --- | --- | --- | --- | --- | --- | --- | --- | --- | --- | --- |
|  | **Window** | **loc.1** | **loc.2** | **loc.3** | **loc.4** | **hap.freq** | **coef** | **se** | **t.stat** | **pval** |
| Geno.2 | 1 | A | T | C | C | 0.2534 | 0.24 | 0.58 | 0.41 | 0.67873 |
| Geno.3 | 1 | A | T | C | T | 0.0221 | 0.92 | 1.41 | 0.65 | 0.51564 |
| Geno.4 | 1 | A | T | G | C | 0.1313 | 2.00 | 0.69 | 2.91 | 0.00382 |
| Geno.6 | 1 | T | G | G | C | 0.0748 | -2.62 | 0.95 | -2.75 | 0.00616 |
| Geno.7 | 1 | T | T | C | C | 0.0781 | 1.36 | 0.86 | 1.58 | 0.11430 |
| Geno.rare | 1 | * | * | * | * | 0.0012 | -20.89 | 6.37 | -3.28 | 0.00113 |
| haplo.base | 1 | A | G | G | C | 0.4390 | NA | NA | NA | NA |
| Geno.5 | 2 | T | C | C | G | 0.3288 | 1.04 | 0.49 | 2.13 | 0.03342 |
| Geno.61 | 2 | T | C | T | G | 0.0214 | 1.56 | 1.45 | 1.07 | 0.28307 |
| Geno.71 | 2 | T | G | C | A | 0.1258 | 2.47 | 0.70 | 3.55 | 0.00042 |
| Geno.rare1 | 2 | * | * | * | * | 0.0104 | -4.18 | 2.26 | -1.85 | 0.06480 |
| haplo.base1 | 2 | G | G | C | G | 0.5136 | NA | NA | NA | NA |
| Geno.21 | 3 | C | C | G | G | 0.3301 | 0.18 | 0.51 | 0.36 | 0.72077 |
| Geno.31 | 3 | C | T | G | G | 0.0222 | 0.46 | 1.42 | 0.32 | 0.74598 |
| Geno.51 | 3 | G | C | A | G | 0.1270 | 1.65 | 0.70 | 2.34 | 0.01960 |
| Geno.72 | 3 | G | C | G | G | 0.1212 | -3.20 | 0.70 | -4.53 | 0.00001 |
| Geno.rare2 | 3 | * | * | * | * | 0.0025 | -6.15 | 4.42 | -1.39 | 0.16502 |
| haplo.base2 | 3 | G | C | G | A | 0.3970 | NA | NA | NA | NA |
| Geno.32 | 4 | C | A | G | T | 0.1274 | 2.36 | 0.73 | 3.26 | 0.00122 |
| Geno.52 | 4 | C | G | A | T | 0.3962 | 0.71 | 0.49 | 1.46 | 0.14637 |
| Geno.10 | 4 | T | G | G | T | 0.0221 | 1.39 | 1.45 | 0.96 | 0.33866 |
| Geno.rare3 | 4 | * | * | * | * | 0.0104 | 0.06 | 2.23 | 0.03 | 0.97919 |
| haplo.base3 | 4 | C | G | G | T | 0.4438 | NA | NA | NA | NA |
| Geno.33 | 5 | A | G | T | G | 0.1269 | 2.26 | 0.72 | 3.13 | 0.00185 |
| Geno.62 | 5 | G | A | T | G | 0.3951 | 0.59 | 0.49 | 1.22 | 0.22353 |
| Geno.rare4 | 5 | * | * | * | * | 0.0115 | 1.49 | 2.10 | 0.71 | 0.47789 |
| haplo.base4 | 5 | G | G | T | G | 0.4666 | NA | NA | NA | NA |
| Geno.63 | 7 | T | G | T | C | 0.1582 | 2.04 | 0.61 | 3.31 | 0.00101 |
| Geno.rare6 | 7 | * | * | * | * | 0.0127 | 2.37 | 1.97 | 1.20 | 0.22976 |
| haplo.base6 | 7 | T | G | T | T | 0.8291 | NA | NA | NA | NA |
| Geno.35 | 8 | G | T | C | C | 0.1606 | 1.52 | 0.58 | 2.63 | 0.00888 |
| Geno.64 | 8 | G | T | T | T | 0.0810 | -5.37 | 0.76 | -7.03 | 8.41E-12 |
| Geno.rare7 | 8 | * | * | * | * | 0.0032 | 10.05 | 4.26 | 2.36 | 0.01883 |
| haplo.base7 | 8 | G | T | T | C | 0.7552 | NA | NA | NA | NA |
| Geno.36 | 9 | T | C | C | T | 0.1586 | 1.37 | 0.60 | 2.28 | 0.02327 |
| Geno.73 | 9 | T | T | T | T | 0.0797 | -5.53 | 0.81 | -6.79 | 3.76E-11 |
| Geno.rare8 | 9 | * | * | * | * | 0.0056 | 3.67 | 3.38 | 1.09 | 0.27814 |
| haplo.base8 | 9 | T | T | C | T | 0.7560 | NA | NA | NA | NA |
| Geno.22 | 10 | C | C | T | T | 0.1613 | 1.59 | 0.59 | 2.68 | 0.00761 |
| Geno.53 | 10 | T | C | T | C | 0.0737 | 0.42 | 0.80 | 0.52 | 0.60085 |
| Geno.8 | 10 | T | T | T | T | 0.0819 | -5.28 | 0.76 | -6.94 | 1.46E-11 |
| Geno.rare9 | 10 | * | * | * | * | 0.0023 | 0.72 | 4.31 | 0.17 | 0.86823 |
| haplo.base9 | 10 | T | C | T | T | 0.6807 | NA | NA | NA | NA |

hap.freq: haplotype frequency; coef: coefficient; se: standard error; t.stat: test statistic; p-val: haplotype p-value; ^a^ Box-Cox transformed data.

Table S11. Haplotype-based association summary of significant windows with TG in NHWs (n=623)

| **TG^a^** | | | | | | | | | | |
| --- | --- | --- | --- | --- | --- | --- | --- | --- | --- | --- |
|  | **Window** | **loc.1** | **loc.2** | **loc.3** | **loc.4** | **hap.freq** | **coef** | **se** | **t.stat** | **pval** |
| Geno.3 | 1 | A | T | C | C | 0.2597 | -0.093 | 0.030 | -3.063 | 0.00229 |
| Geno.4 | 1 | A | T | C | T | 0.0212 | -0.125 | 0.077 | -1.614 | 0.10696 |
| Geno.5 | 1 | A | T | G | C | 0.1191 | -0.021 | 0.037 | -0.563 | 0.57379 |
| Geno.7 | 1 | T | G | G | C | 0.0815 | -0.047 | 0.048 | -0.979 | 0.32783 |
| Geno.8 | 1 | T | T | C | C | 0.0767 | -0.074 | 0.046 | -1.602 | 0.10978 |
| Geno.rare | 1 | * | * | * | * | 0.0018 | 0.680 | 0.387 | 1.755 | 0.07969 |
| haplo.base | 1 | A | G | G | C | 0.4400 | NA | NA | NA | NA |
| Geno.51 | 2 | T | C | C | G | 0.3351 | -0.079 | 0.025 | -3.107 | 0.00198 |
| Geno.6 | 2 | T | C | T | G | 0.0208 | -0.119 | 0.078 | -1.523 | 0.12829 |
| Geno.71 | 2 | T | G | C | A | 0.1134 | -0.008 | 0.038 | -0.201 | 0.84085 |
| Geno.rare1 | 2 | * | * | * | * | 0.0106 | -0.091 | 0.115 | -0.796 | 0.42648 |
| haplo.base1 | 2 | G | G | C | G | 0.5201 | NA | NA | NA | NA |
| Geno.2 | 3 | C | C | G | G | 0.3364 | -0.083 | 0.027 | -3.082 | 0.00215 |
| Geno.31 | 3 | C | T | G | G | 0.0212 | -0.130 | 0.078 | -1.667 | 0.09597 |
| Geno.52 | 3 | G | C | A | G | 0.1148 | -0.019 | 0.038 | -0.486 | 0.62712 |
| Geno.72 | 3 | G | C | G | G | 0.1220 | -0.047 | 0.038 | -1.261 | 0.20779 |
| Geno.rare2 | 3 | * | * | * | * | 0.0019 | -0.439 | 0.275 | -1.598 | 0.11047 |
| haplo.base2 | 3 | G | C | G | A | 0.4037 | NA | NA | NA | NA |
| Geno.33 | 5 | A | G | T | G | 0.1146 | 0.048 | 0.038 | 1.257 | 0.20924 |
| Geno.61 | 5 | G | A | T | G | 0.4015 | 0.072 | 0.025 | 2.902 | 0.00384 |
| Geno.rare4 | 5 | * | * | * | * | 0.0129 | -0.003 | 0.102 | -0.026 | 0.97936 |
| haplo.base4 | 5 | G | G | T | G | 0.4710 | NA | NA | NA | NA |

hap.freq: haplotype frequency; coef: coefficient; se: standard error; t.stat: test statistic; p-val: haplotype p-value; ^a^ Box-Cox transformed data.

Table S12. Haplotype-based association summary of significant windows with HDL-C in NHWs (n=623)

| **HDL-C^a^** | | | | | | | | | | |
| --- | --- | --- | --- | --- | --- | --- | --- | --- | --- | --- |
|  | **Window** | **loc.1** | **loc.2** | **loc.3** | **loc.4** | **hap.freq** | **coef** | **se** | **t.stat** | **pval** |
| Geno.37 | 12 | T | C | C | C | 0.0814 | 0.034 | 0.019 | 1.765 | 0.07798 |
| Geno.rare11 | 12 | * | * | * | * | 0.0130 | 0.096 | 0.050 | 1.934 | 0.05354 |
| haplo.base11 | 12 | T | C | C | T | 0.9057 | NA | NA | NA | NA |

hap.freq: haplotype frequency; coef: coefficient; se: standard error; t.stat: test statistic; p-val: haplotype p-value; ^a^ Box-Cox transformed data.

Table S13. 4-SNPs window haplotype-based association results for lipid traits in African Blacks (n=788)

|  |  |  |  |  | **LDL-C^a^** | | | **ApoB^a^** | | | **TG^a^** | | | **HDL-C^a^** | | |
| --- | --- | --- | --- | --- | --- | --- | --- | --- | --- | --- | --- | --- | --- | --- | --- | --- |
| **wind** | **w.snp** | **Location** | **Genotype** | **MAF** | **Beta** | **p_value** | **hap_P** | **Beta** | **p_value** | **hap_P** | **Beta** | **p_value** | **hap_P** | **Beta** | **p_value** | **hap_P** |
| 1 | APOE73/rs1081101 | 5'flanking | CC/CT/TT | 0.0611 | -0.62 | 0.1110 | **0.0217** | 0.65 | 0.5488 | 0.2732 | 0.038 | 0.0093 | **0.0353** | 0.30 | 0.4273 | 0.5416 |
| 1 | APOE173 | 5'flanking | AA/GA | 0.0020 | -1.14 | 0.5979 | NA | 3.20 | 0.5923 | NA | 0.037 | 0.7028 | NA | -2.10 | 0.3206 | NA |
| 1 | APOE308/rs769445 | 5'flanking | CC/TC | 0.0072 | -0.37 | 0.7576 | NA | 1.87 | 0.5514 | NA | 0.056 | 0.2065 | NA | -1.06 | 0.3428 | NA |
| 1 | APOE560/rs449647 | 5'flanking | AA/AT/TT | 0.3663 | 0.58 | 0.0031 | NA | 0.92 | 0.0951 | NA | -0.001 | 0.8482 | NA | -0.18 | 0.3564 | NA |
| 2 | APOE173 | 5'flanking | AA/GA | 0.0020 | -1.14 | 0.5979 | **0.0097** | 3.20 | 0.5923 | 0.2158 | 0.037 | 0.7028 | 0.4467 | -2.10 | 0.3206 | 0.1090 |
| 2 | APOE308/rs769445 | 5'flanking | CC/TC | 0.0072 | -0.37 | 0.7576 | NA | 1.87 | 0.5514 | NA | 0.056 | 0.2065 | NA | -1.06 | 0.3428 | NA |
| 2 | APOE560/rs449647 | 5'flanking | AA/AT/TT | 0.3663 | 0.58 | 0.0031 | NA | 0.92 | 0.0951 | NA | -0.001 | 0.8482 | NA | -0.18 | 0.3564 | NA |
| 2 | APOE618 | 5'flanking | GC/GG | 0.0006 | -2.85 | 0.4432 | NA | 4.15 | 0.6876 | NA | 0.025 | 0.8560 | NA | -12.25 | 0.0008 | NA |
| 3 | APOE308rs769445 | 5'flanking | CC/TC | 0.0072 | -0.37 | 0.7576 | **0.0066** | 1.87 | 0.5514 | 0.3232 | 0.056 | 0.2065 | 0.6938 | -1.06 | 0.3428 | 0.5433 |
| 3 | APOE560 | 5'flanking | AA/AT/TT | 0.3663 | 0.58 | 0.0031 | NA | 0.92 | 0.0951 | NA | -0.001 | 0.8482 | NA | -0.18 | 0.3564 | NA |
| 3 | APOE618 | 5'flanking | GC/GG | 0.0006 | -2.85 | 0.4432 | NA | 4.15 | 0.6876 | NA | 0.025 | 0.8560 | NA | -12.25 | 0.0008 | NA |
| 3 | APOE624/rs769446 | 5'flanking | TC/TT | 0.0077 | -2.86 | 0.0214 | NA | -4.19 | 0.2037 | NA | 0.005 | 0.9096 | NA | 0.65 | 0.5687 | NA |
| 4 | APOE560 | 5'flanking | AA/AT/TT | 0.3663 | 0.58 | 0.0031 | **0.0034** | 0.92 | 0.0951 | 0.2611 | -0.001 | 0.8482 | 0.8396 | -0.18 | 0.3564 | 0.0515 |
| 4 | APOE618 | 5'flanking | GC/GG | 0.0006 | -2.85 | 0.4432 | NA | 4.15 | 0.6876 | NA | 0.025 | 0.8560 | NA | -12.25 | 0.0008 | NA |
| 4 | APOE624/rs769446 | 5'flanking | TC/TT | 0.0077 | -2.86 | 0.0214 | NA | -4.19 | 0.2037 | NA | 0.005 | 0.9096 | NA | 0.65 | 0.5687 | NA |
| 4 | APOE832/rs405509 | 5'flanking | GG/GT/TT | 0.2561 | 0.60 | 0.0044 | NA | 0.66 | 0.2611 | NA | 0.003 | 0.7457 | NA | -0.19 | 0.3612 | NA |
| 5 | APOE618 | 5'flanking | GC/GG | 0.0006 | -2.85 | 0.4432 | **0.0034** | 4.15 | 0.6876 | 0.2682 | 0.025 | 0.8560 | 0.5536 | -12.25 | 0.0008 | 0.2741 |
| 5 | APOE624/rs769446 | 5'flanking | TC/TT | 0.0077 | -2.86 | 0.0214 | NA | -4.19 | 0.2037 | NA | 0.005 | 0.9096 | NA | 0.65 | 0.5687 | NA |
| 5 | APOE832/rs405509 | 5'flanking | GG/GT/TT | 0.2561 | 0.60 | 0.0044 | NA | 0.66 | 0.2611 | NA | 0.003 | 0.7457 | NA | -0.19 | 0.3612 | NA |
| 5 | APOE1109/rs9282609 | splice site | CC/TC/TT | 0.0415 | 0.05 | 0.9183 | NA | 1.38 | 0.2768 | NA | 0.023 | 0.1814 | NA | 0.61 | 0.1788 | NA |
| 6 | APOE624/rs769446 | 5'flanking | TC/TT | 0.0077 | -2.86 | 0.0214 | **0.0124** | -4.19 | 0.2037 | 0.1856 | 0.005 | 0.9096 | 0.1645 | 0.65 | 0.5687 | 0.4314 |
| 6 | APOE832/rs405509 | 5'flanking | GG/GT/TT | 0.2561 | 0.60 | 0.0044 | NA | 0.66 | 0.2611 | NA | 0.003 | 0.7457 | NA | -0.19 | 0.3612 | NA |
| 6 | APOE1109/rs9282609 | splice site | CC/TC/TT | 0.0415 | 0.05 | 0.9183 | NA | 1.38 | 0.2768 | NA | 0.023 | 0.1814 | NA | 0.61 | 0.1788 | NA |
| 6 | APOE1163/rs440446 | intron1 | CC/CG/GG | 0.1004 | 0.44 | 0.1791 | NA | 2.30 | 0.0109 | NA | 0.023 | 0.0537 | NA | -0.54 | 0.0946 | NA |
| 7 | APOE832/rs405509 | 5'flanking | GG/GT/TT | 0.2561 | 0.60 | 0.0044 | 0.0895 | 0.66 | 0.2611 | 0.1322 | 0.003 | 0.7457 | 0.1752 | -0.19 | 0.3612 | 0.3319 |
| 7 | APOE1109/rs9282609 | splice site | CC/TC/TT | 0.0415 | 0.05 | 0.9183 | NA | 1.38 | 0.2768 | NA | 0.023 | 0.1814 | NA | 0.61 | 0.1788 | NA |
| 7 | APOE1163/rs440446 | intron1 | CC/CG/GG | 0.1004 | 0.44 | 0.1791 | NA | 2.30 | 0.0109 | NA | 0.023 | 0.0537 | NA | -0.54 | 0.0946 | NA |
| 7 | APOE1231 | intron1 | GA/GG | 0.0125 | -0.71 | 0.4139 | NA | -1.71 | 0.4748 | NA | -0.030 | 0.3441 | NA | -0.27 | 0.7550 | NA |
| 8 | APOE1109/rs9282609 | splice site | CC/TC/TT | 0.0415 | 0.05 | 0.9183 | 0.0781 | 1.38 | 0.2768 | 0.0897 | 0.023 | 0.1814 | 0.0587 | 0.61 | 0.1788 | 0.2063 |
| 8 | APOE1163/rs440446 | intron1 | CC/CG/GG | 0.1004 | 0.44 | 0.1791 | NA | 2.30 | 0.0109 | NA | 0.023 | 0.0537 | NA | -0.54 | 0.0946 | NA |
| 8 | APOE1231 | intron1 | GA/GG | 0.0125 | -0.71 | 0.4139 | NA | -1.71 | 0.4748 | NA | -0.030 | 0.3441 | NA | -0.27 | 0.7550 | NA |
| 8 | APOE1279/rs877973 | intron1 | AA/CA/CC | 0.0597 | -0.15 | 0.7208 | NA | -0.45 | 0.6847 | NA | -0.029 | 0.0513 | NA | 0.51 | 0.1981 | NA |
| 9 | APOE1163/rs440446 | intron1 | CC/CG/GG | 0.1004 | 0.44 | 0.1791 | 0.4838 | 2.30 | 0.0109 | 0.1435 | 0.023 | 0.0537 | 0.0889 | -0.54 | 0.0946 | 0.1736 |
| 9 | APOE1231 | intron1 | GA/GG | 0.0125 | -0.71 | 0.4139 | NA | -1.71 | 0.4748 | NA | -0.030 | 0.3441 | NA | -0.27 | 0.7550 | NA |
| 9 | APOE1279/rs877973 | intron1 | AA/CA/CC | 0.0597 | -0.15 | 0.7208 | NA | -0.45 | 0.6847 | NA | -0.029 | 0.0513 | NA | 0.51 | 0.1981 | NA |
| 9 | APOE1539/rs184686013 | iintron1 | AA/AG/GG | 0.0086 | -1.03 | 0.2862 | NA | 0.23 | 0.9330 | NA | 0.002 | 0.9546 | NA | 1.35 | 0.1568 | NA |
| 10 | APOE1231 | intron1 | GA/GG | 0.0125 | -0.71 | 0.4139 | 0.5335 | -1.71 | 0.4748 | 0.8522 | -0.030 | 0.3441 | 0.1782 | -0.27 | 0.7550 | 0.5938 |
| 10 | APOE1279/rs877973 | intron1 | AA/CA/CC | 0.0597 | -0.15 | 0.7208 | NA | -0.45 | 0.6847 | NA | -0.029 | 0.0513 | NA | 0.51 | 0.1981 | NA |
| 10 | APOE1539/rs184686013 | iintron1 | AA/AG/GG | 0.0086 | -1.03 | 0.2862 | NA | 0.23 | 0.9330 | NA | 0.002 | 0.9546 | NA | 1.35 | 0.1568 | NA |
| 10 | APOE2072/rs189660912 | intron 2 | GA/GG | 0.0079 | -0.50 | 0.6433 | NA | 0.70 | 0.8149 | NA | 0.009 | 0.8229 | NA | -0.40 | 0.7066 | NA |
| 11 | APOE1279/rs877973 | intron1 | AA/CA/CC | 0.0597 | -0.15 | 0.7208 | **0.0365** | -0.45 | 0.6847 | 0.7508 | -0.029 | 0.0513 | 0.1112 | 0.51 | 0.1981 | 0.4347 |
| 11 | APOE1539/rs184686013 | intron1 | AA/AG/GG | 0.0086 | -1.03 | 0.2862 | NA | 0.23 | 0.9330 | NA | 0.002 | 0.9546 | NA | 1.35 | 0.1568 | NA |
| 11 | APOE2072/rs189660912 | intron 2 | GA/GG | 0.0079 | -0.50 | 0.6433 | NA | 0.70 | 0.8149 | NA | 0.009 | 0.8229 | NA | -0.40 | 0.7066 | NA |
| 11 | APOE2269/rs61357706 | intron 2 | GA/GG | 0.0169 | -2.05 | 0.0064 | NA | -2.56 | 0.2201 | NA | 0.040 | 0.1489 | NA | 0.77 | 0.2912 | NA |
| 12 | APOE1539/rs184686013 | intron1 | AA/AG/GG | 0.0086 | -1.03 | 0.2862 | **0.0294** | 0.23 | 0.9330 | 0.4195 | 0.002 | 0.9546 | 0.5628 | 1.35 | 0.1568 | 0.6654 |
| 12 | APOE2072/rs189660912 | intron 2 | GA/GG | 0.0079 | -0.50 | 0.6433 | NA | 0.70 | 0.8149 | NA | 0.009 | 0.8229 | NA | -0.40 | 0.7066 | NA |
| 12 | APOE2269/rs61357706 | intron 2 | GA/GG | 0.0169 | -2.05 | 0.0064 | NA | -2.56 | 0.2201 | NA | 0.040 | 0.1489 | NA | 0.77 | 0.2912 | NA |
| 12 | APOE2440/rs769450 | intron 2 | AA/AG/GG | 0.3870 | 0.20 | 0.3409 | NA | -0.50 | 0.3725 | NA | -0.003 | 0.7396 | NA | -0.02 | 0.9394 | NA |
| 13 | APOE2072/rs189660912 | intron 2 | GA/GG | 0.0079 | -0.50 | 0.6433 | **0.0244** | 0.70 | 0.8149 | 0.1158 | 0.009 | 0.8229 | 0.4828 | -0.40 | 0.7066 | 0.9467 |
| 13 | APOE2269/rs61357706 | intron 2 | GA/GG | 0.0169 | -2.05 | 0.0064 | NA | -2.56 | 0.2201 | NA | 0.040 | 0.1489 | NA | 0.77 | 0.2912 | NA |
| 13 | APOE2440/rs769450 | intron 2 | AA/AG/GG | 0.3870 | 0.20 | 0.3409 | NA | -0.50 | 0.3725 | NA | -0.003 | 0.7396 | NA | -0.02 | 0.9394 | NA |
| 13 | APOE2544/rs115299243 | intron 2 | AA/GA/GG | 0.0190 | -2.34 | 0.0017 | NA | -4.01 | 0.0524 | NA | 0.061 | 0.0470 | NA | -0.54 | 0.4827 | NA |
| 14 | APOE2269/rs61357706 | intron 2 | GA/GG | 0.0169 | -2.05 | 0.0064 | **0.0260** | -2.56 | 0.2201 | 0.1078 | 0.040 | 0.1489 | 0.3794 | 0.77 | 0.2912 | 0.7629 |
| 14 | APOE2440/rs769450 | intron 2 | AA/AG/GG | 0.3870 | 0.20 | 0.3409 | NA | -0.50 | 0.3725 | NA | -0.003 | 0.7396 | NA | -0.02 | 0.9394 | NA |
| 14 | APOE2544/rs115299243 | intron 2 | AA/GA/GG | 0.0190 | -2.34 | 0.0017 | NA | -4.01 | 0.0524 | NA | 0.061 | 0.0470 | NA | -0.54 | 0.4827 | NA |
| 14 | APOE3673/rs769453 | intron 3 | CC/GC | 0.0066 | -0.37 | 0.7649 | NA | 2.02 | 0.5386 | NA | 0.037 | 0.4242 | NA | -1.10 | 0.3457 | NA |
| 15 | APOE2440/rs769450 | intron 2 | AA/AG/GG | 0.3870 | 0.20 | 0.3409 | **0.0022** | -0.50 | 0.3725 | 0.3120 | -0.003 | 0.7396 | 0.1502 | -0.02 | 0.9394 | 0.7187 |
| 15 | APOE2544/rs115299243 | intron 2 | AA/GA/GG | 0.0190 | -2.34 | 0.0017 | NA | -4.01 | 0.0524 | NA | 0.061 | 0.0470 | NA | -0.54 | 0.4827 | NA |
| 15 | APOE3673/rs769453 | intron 3 | CC/GC | 0.0066 | -0.37 | 0.7649 | NA | 2.02 | 0.5386 | NA | 0.037 | 0.4242 | NA | -1.10 | 0.3457 | NA |
| 15 | APOE3937/rs429358 | exon 4 (Cys 130 Arg) | CC/CT/TT | 0.2656 | 0.46 | 0.0317 | NA | 0.05 | 0.9371 | NA | -0.008 | 0.3075 | NA | -0.14 | 0.5074 | NA |
| 16 | APOE2544/rs115299243 | intron 2 | AA/GA/GG | 0.0190 | -2.34 | 0.0017 | **0.0023** | -4.01 | 0.0524 | 0.3120 | 0.061 | 0.0470 | 0.1234 | -0.54 | 0.4827 | 0.5466 |
| 16 | APOE3673/rs769453 | intron 3 | CC/GC | 0.0066 | -0.37 | 0.7649 | NA | 2.02 | 0.5386 | NA | 0.037 | 0.4242 | NA | -1.10 | 0.3457 | NA |
| 16 | APOE3937/rs429358 | exon 4 (Cys 130 Arg) | CC/CT/TT | 0.2656 | 0.46 | 0.0317 | NA | 0.05 | 0.9371 | NA | -0.008 | 0.3075 | NA | -0.14 | 0.5074 | NA |
| 16 | APOE4036/rs769455 | exon 4 (Arg 163 Cys) | CC/TC/TT | 0.0200 | -2.23 | 0.0009 | NA | -3.45 | 0.0664 | NA | 0.056 | 0.0372 | NA | -0.46 | 0.4827 | NA |
| 17 | APOE3673/rs769453 | intron 3 | CC/GC | 0.0066 | -0.37 | 0.7649 | **3.41E-08** | 2.02 | 0.5386 | 0.0660 | 0.037 | 0.4242 | 0.1297 | -1.10 | 0.3457 | 0.2975 |
| 17 | APOE3937/rs429358 | exon 4 (Cys 130 Arg) | CC/CT/TT | 0.2656 | 0.46 | 0.0317 | NA | 0.05 | 0.9371 | NA | -0.008 | 0.3075 | NA | -0.14 | 0.5074 | NA |
| 17 | APOE4036/rs769455 | exon 4 (Arg 163 Cys) | CC/TC/TT | 0.0200 | -2.23 | 0.0009 | NA | -3.45 | 0.0664 | NA | 0.056 | 0.0372 | NA | -0.46 | 0.4827 | NA |
| 17 | APOE4075/rs7412 | exon 4 (Arg 176 Cys) | AA/GA/GG | 0.0605 | -2.05 | 5.35E-07 | NA | -2.35 | 0.0356 | NA | -0.018 | 0.2376 | NA | 0.75 | 0.0661 | NA |
| 18 | APOE3937/rs429358 | exon 4 (Cys 130 Arg) | CC/CT/TT | 0.2656 | 0.46 | 0.0317 | **9.58E-09** | 0.05 | 0.9371 | **0.0350** | -0.008 | 0.3075 | 0.0834 | -0.14 | 0.5074 | 0.2806 |
| 18 | APOE4036/rs769455 | exon 4 (Arg 163 Cys) | CC/TC/TT | 0.0200 | -2.23 | 0.0009 | NA | -3.45 | 0.0664 | NA | 0.056 | 0.0372 | NA | -0.46 | 0.4827 | NA |
| 18 | APOE4075/rs7412 | exon 4 (Arg 176 Cys) | AA/GA/GG | 0.0605 | -2.05 | 5.35E-07 | NA | -2.35 | 0.0356 | NA | -0.018 | 0.2376 | NA | 0.75 | 0.0661 | NA |
| 18 | APOE4569 | exon 4 (3'UTR) | GG/GT | 0.0007 | 8.87 | 0.0173 | NA | 14.25 | 0.1684 | NA | 0.073 | 0.5976 | NA | 5.29 | 0.1497 | NA |
| 19 | APOE4036/rs769455 | exon 4 (Arg 163 Cys) | CC/TC/TT | 0.0200 | -2.23 | 0.0009 | **8.86E-09** | -3.45 | 0.0664 | **0.0376** | 0.056 | 0.0372 | 0.1379 | -0.46 | 0.4827 | 0.2470 |
| 19 | APOE4075/rs7412 | exon 4 (Arg 176 Cys) | AA/GA/GG | 0.0605 | -2.05 | 5.35E-07 | NA | -2.35 | 0.0356 | NA | -0.018 | 0.2376 | NA | 0.75 | 0.0661 | NA |
| 19 | APOE4569 | exon 4 (3'UTR) | GG/GT | 0.0007 | 8.87 | 0.0173 | NA | 14.25 | 0.1684 | NA | 0.073 | 0.5976 | NA | 5.29 | 0.1497 | NA |
| 19 | APOE5223 | 3'flanking | CC/CG | 0.0051 | -2.25 | 0.0874 | NA | -2.54 | 0.5166 | NA | 0.006 | 0.9078 | NA | 0.39 | 0.7630 | NA |
| 20 | APOE4075/rs7412 | exon 4 (Arg 176 Cys) | AA/GA/GG | 0.0605 | -2.05 | 5.35E-07 | **8.23E-06** | -2.35 | 0.0356 | 0.1962 | -0.018 | 0.2376 | 0.3979 | 0.75 | 0.0661 | 0.2851 |
| 20 | APOE4569 | exon 4 (3'UTR) | GG/GT | 0.0007 | 8.87 | 0.0173 | NA | 14.25 | 0.1684 | NA | 0.073 | 0.5976 | NA | 5.29 | 0.1497 | NA |
| 20 | APOE5223 | 3'flanking | CC/CG | 0.0051 | -2.25 | 0.0874 | NA | -2.54 | 0.5166 | NA | 0.006 | 0.9078 | NA | 0.39 | 0.7630 | NA |
| 20 | APOE5231 | 3'flanking | GG/GT/TT | 0.0270 | -0.10 | 0.8587 | NA | -0.82 | 0.6036 | NA | -0.023 | 0.2836 | NA | -0.18 | 0.7645 | NA |

wind: 4-SNPs haplotype window; w.snps: SNPs that were included in each window; p-value: single-site p-value; hap-P: haplotype global p-value; ^a^Box-Cox transformed data.

Table S14. Haplotype-based association summary of significant windows with LDL-C in African Blacks (n=788)

| **LDL-C^a^** | | | | | | | | | | |
| --- | --- | --- | --- | --- | --- | --- | --- | --- | --- | --- |
|  | Window | loc.1 | loc.2 | loc.3 | loc.4 | hap.freq | coef | se | t.stat | pval |
| Geno.2 | 1 | C | A | C | T | 0.3649 | 0.53 | 0.20 | 2.65 | 0.00815 |
| Geno.6 | 1 | T | A | C | A | 0.0614 | -0.36 | 0.40 | -0.91 | 0.36339 |
| Geno.rare | 1 | * | * | * | * | 0.0067 | -0.25 | 1.29 | -0.20 | 0.84412 |
| haplo.base | 1 | C | A | C | A | 0.5670 | NA | NA | NA | NA |
| Geno.4 | 2 | A | C | T | G | 0.3646 | 0.59 | 0.19 | 3.01 | 0.00266 |
| Geno.rare1 | 2 | * | * | * | * | 0.0073 | -0.40 | 1.33 | -0.30 | 0.76355 |
| haplo.base1 | 2 | A | C | A | G | 0.6280 | NA | NA | NA | NA |
| Geno.61 | 3 | C | T | G | T | 0.3631 | 0.54 | 0.20 | 2.79 | 0.00547 |
| Geno.rare2 | 3 | * | * | * | * | 0.0130 | -1.01 | 0.92 | -1.10 | 0.27269 |
| haplo.base2 | 3 | C | A | G | T | 0.6239 | NA | NA | NA | NA |
| Geno.3 | 4 | A | G | T | T | 0.0422 | 0.50 | 0.50 | 1.01 | 0.31120 |
| Geno.62 | 4 | T | G | T | G | 0.1566 | 0.43 | 0.28 | 1.54 | 0.12379 |
| Geno.7 | 4 | T | G | T | T | 0.2142 | 0.67 | 0.23 | 2.88 | 0.00406 |
| Geno.rare3 | 4 | * | * | * | * | 0.0072 | -2.53 | 1.26 | -2.01 | 0.04529 |
| haplo.base3 | 4 | A | G | T | G | 0.5798 | NA | NA | NA | NA |
| Geno.5 | 5 | G | T | G | T | 0.0414 | 0.24 | 0.46 | 0.53 | 0.59901 |
| Geno.63 | 5 | G | T | T | C | 0.2571 | 0.58 | 0.21 | 2.76 | 0.00592 |
| Geno.rare4 | 5 | * | * | * | * | 0.0071 | -2.71 | 1.27 | -2.13 | 0.03354 |
| haplo.base4 | 5 | G | T | G | C | 0.6944 | NA | NA | NA | NA |
| Geno.51 | 6 | T | G | T | G | 0.0421 | 0.19 | 0.46 | 0.41 | 0.67890 |
| Geno.64 | 6 | T | T | C | C | 0.0984 | 0.65 | 0.32 | 2.00 | 0.04579 |
| Geno.71 | 6 | T | T | C | G | 0.1582 | 0.53 | 0.26 | 2.01 | 0.04437 |
| Geno.rare5 | 6 | * | * | * | * | 0.0139 | -1.83 | 0.89 | -2.05 | 0.04074 |
| haplo.base5 | 6 | T | G | C | G | 0.6875 | NA | NA | NA | NA |
| Geno.25 | 11 | A | A | G | G | 0.0563 | -0.19 | 0.41 | -0.47 | 0.64066 |
| Geno.66 | 11 | C | A | G | A | 0.0159 | -2.06 | 0.79 | -2.59 | 0.00971 |
| Geno.rare10 | 11 | * | * | * | * | 0.0163 | -0.93 | 0.77 | -1.21 | 0.22486 |
| haplo.base10 | 11 | C | A | G | G | 0.9114 | NA | NA | NA | NA |
| Geno.32 | 12 | A | G | A | G | 0.0168 | -2.00 | 0.75 | -2.69 | 0.00738 |
| Geno.43 | 12 | A | G | G | A | 0.3863 | 0.09 | 0.20 | 0.42 | 0.67194 |
| Geno.rare11 | 12 | * | * | * | * | 0.0155 | -0.81 | 0.74 | -1.09 | 0.27669 |
| haplo.base11 | 12 | A | G | G | G | 0.5814 | NA | NA | NA | NA |
| Geno.33 | 13 | G | A | G | G | 0.0192 | -1.94 | 0.67 | -2.88 | 0.00405 |
| Geno.44 | 13 | G | G | A | A | 0.3859 | 0.11 | 0.20 | 0.53 | 0.59958 |
| Geno.rare12 | 13 | * | * | * | * | 0.0080 | -0.51 | 1.07 | -0.47 | 0.63749 |
| haplo.base12 | 13 | G | G | G | A | 0.5869 | NA | NA | NA | NA |
| Geno.26 | 14 | A | G | G | C | 0.0192 | -1.94 | 0.67 | -2.87 | 0.00418 |
| Geno.34 | 14 | G | A | A | C | 0.3861 | 0.11 | 0.20 | 0.57 | 0.57101 |
| Geno.rare13 | 14 | * | * | * | * | 0.0060 | -0.34 | 1.23 | -0.28 | 0.77983 |
| haplo.base13 | 14 | G | G | A | C | 0.5887 | NA | NA | NA | NA |
| Geno.45 | 15 | G | A | C | C | 0.2669 | 0.22 | 0.23 | 0.93 | 0.35474 |
| Geno.54 | 15 | G | A | C | T | 0.3268 | -0.42 | 0.23 | -1.81 | 0.07024 |
| Geno.9 | 15 | G | G | C | T | 0.0178 | -2.37 | 0.75 | -3.15 | 0.00171 |
| Geno.rare14 | 15 | * | * | * | * | 0.0071 | -0.45 | 1.17 | -0.38 | 0.70129 |
| haplo.base14 | 15 | A | A | C | T | 0.3813 | NA | NA | NA | NA |
| Geno.11 | 16 | A | C | C | C | 0.2675 | 0.40 | 0.21 | 1.91 | 0.05625 |
| Geno.67 | 16 | G | C | T | T | 0.0194 | -2.11 | 0.67 | -3.16 | 0.00167 |
| Geno.rare15 | 16 | * | * | * | * | 0.0060 | -0.37 | 1.31 | -0.28 | 0.77767 |
| haplo.base15 | 16 | A | C | T | C | 0.7072 | NA | NA | NA | NA |
| Geno.12 | 17 | C | C | C | G | 0.2674 | 0.22 | 0.21 | 1.04 | 0.29819 |
| Geno.35 | 17 | C | T | C | A | 0.0581 | -2.05 | 0.40 | -5.08 | 4.65E-07 |
| Geno.55 | 17 | C | T | T | G | 0.0198 | -2.47 | 0.67 | -3.69 | 0.00024 |
| Geno.rare16 | 17 | * | * | * | * | 0.0060 | -0.43 | 1.24 | -0.35 | 0.72988 |
| haplo.base16 | 17 | C | T | C | G | 0.6486 | NA | NA | NA | NA |
| Geno.13 | 18 | C | C | G | G | 0.2669 | 0.19 | 0.21 | 0.92 | 0.35766 |
| Geno.46 | 18 | T | C | A | G | 0.0581 | -2.06 | 0.40 | -5.11 | 4.14E-07 |
| Geno.68 | 18 | T | T | G | G | 0.0198 | -2.47 | 0.67 | -3.69 | 0.00024 |
| haplo.base17 | 18 | T | C | G | G | 0.6545 | NA | NA | NA | NA |
| Geno.14 | 19 | C | A | G | C | 0.0581 | -2.14 | 0.40 | -5.39 | 9.62E-08 |
| Geno.69 | 19 | T | G | G | C | 0.0198 | -2.51 | 0.67 | -3.78 | 0.00017 |
| Geno.rare17 | 19 | * | * | * | * | 0.0059 | -1.35 | 1.28 | -1.06 | 0.28994 |
| haplo.base18 | 19 | C | G | G | C | 0.9162 | NA | NA | NA | NA |
| Geno.27 | 20 | A | G | C | T | 0.0582 | -2.04 | 0.40 | -5.09 | 4.44E-07 |
| Geno.36 | 20 | G | G | C | G | 0.0263 | -0.16 | 0.56 | -0.28 | 0.77761 |
| Geno.rare18 | 20 | * | * | * | * | 0.0059 | -1.25 | 1.37 | -0.91 | 0.36338 |
| haplo.base19 | 20 | G | G | C | T | 0.9097 | NA | NA | NA | NA |
| hap.freq: haplotype frequency; coef: coefficient; se: standard error; t.stat: test statistic; p-val: haplotype p-value; ^a^Box-Cox transformed data. | | | | | | | | | | |

Table S15. Haplotype-based association summary of significant windows with ApoB in African Blacks (n=788)

| **ApoB^a^** | | | | | | | | | | |
| --- | --- | --- | --- | --- | --- | --- | --- | --- | --- | --- |
|  | **Window** | **loc.1** | **loc.2** | **loc.3** | **loc.4** | **hap.freq** | **coef** | **se** | **t.stat** | **pval** |
| Geno.13 | 18 | C | C | G | G | 0.2652 | -0.30 | 0.60 | -0.50 | 0.61601 |
| Geno.47 | 18 | T | C | A | G | 0.0593 | -2.59 | 1.13 | -2.29 | 0.02206 |
| Geno.66 | 18 | T | T | G | G | 0.0198 | -3.83 | 1.90 | -2.01 | 0.04480 |
| haplo.base17 | 18 | T | C | G | G | 0.6551 | NA | NA | NA | NA |
| Geno.14 | 19 | C | A | G | C | 0.0592 | -2.51 | 1.11 | -2.25 | 0.02478 |
| Geno.55 | 19 | T | G | G | C | 0.0198 | -3.80 | 1.91 | -1.99 | 0.04669 |
| Geno.rare17 | 19 | * | * | * | * | 0.0052 | -0.89 | 3.90 | -0.23 | 0.81963 |
| haplo.base18 | 19 | C | G | G | C | 0.9158 | NA | NA | NA | NA |
| hap.freq: haplotype frequency; coef: coefficient; se: standard error; t.stat: test statistic; p-val: haplotype p-value; ^a^Box-Cox transformed data. | | | | | | | | | | |

Table S16. Haplotype-based association summary of significant windows with TG in African Blacks (n=788)

| **TG^a^** | | | | | | | | | | |
| --- | --- | --- | --- | --- | --- | --- | --- | --- | --- | --- |
|  | **Window** | **loc.1** | **loc.2** | **loc.3** | **loc.4** | **hap.freq** | **coef** | **se** | **t.stat** | **pval** |
| Geno.2 | 1 | C | A | C | T | 0.3614 | 0.003 | 0.008 | 0.424 | 0.67171 |
| Geno.6 | 1 | T | A | C | A | 0.0619 | 0.039 | 0.015 | 2.641 | 0.00843 |
| Geno.rare | 1 | * | * | * | * | 0.0067 | 0.060 | 0.045 | 1.313 | 0.18941 |
| haplo.base | 1 | C | A | C | A | 0.5701 | NA | NA | NA | NA |
| hap.freq: haplotype frequency; coef: coefficient; se: standard error; t.stat: test statistic; p-val: haplotype p-value; ^a^Box-Cox transformed data | | | | | | | | | | |
